# Supplementary material for: Neuronal diversity and stereotypy at multiple scales through whole brain morphometry
Source: Nat Commun. 2024 Nov 26;15:10269. doi: 10.1038/s41467-024-54745-6 (PMC11599929; doi:10.1038/s41467-024-54745-6)
Supplement: Supplementary file 1 — Supplementary Information [file 41467_2024_54745_MOESM1_ESM.pdf]

Supplementary Figures

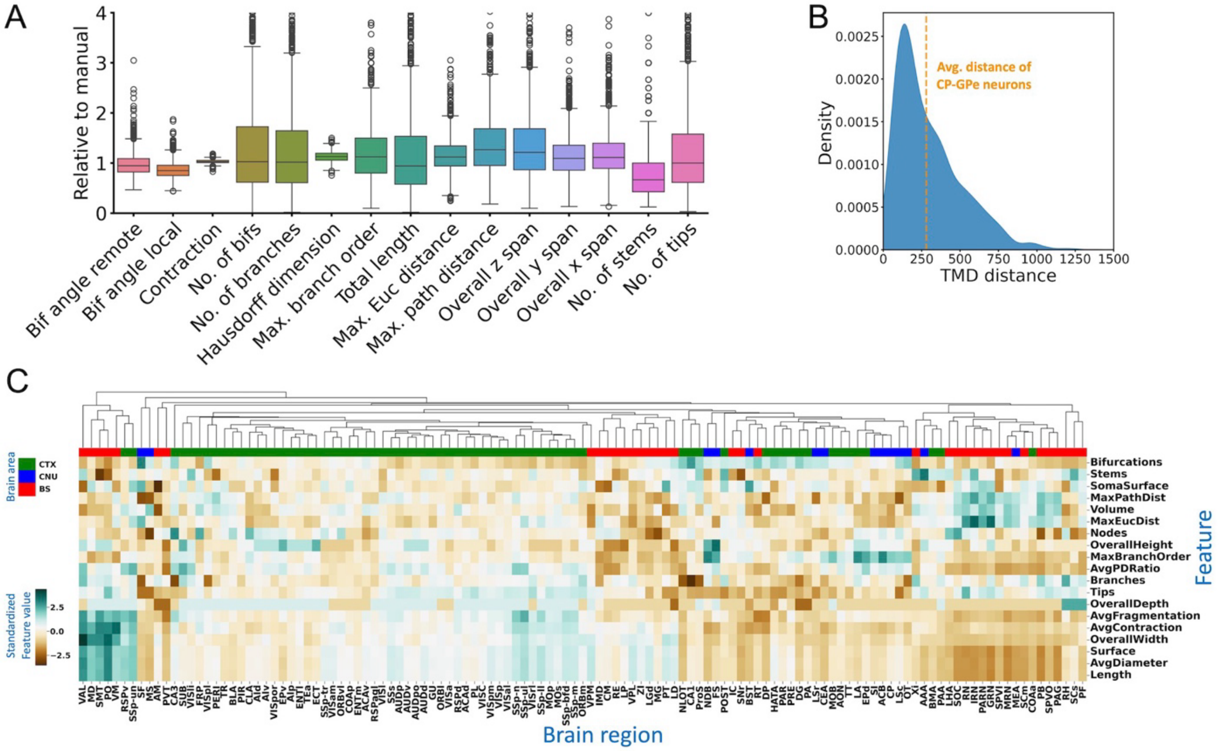

Supplementary Figure S1. **Verification of the auto-traced local morphologies based on L-Measure^{Vaa3D} features.** **A**. Box plots of the 15 L-Measure^{Vaa3D} features. We computed the relative feature values by dividing automated reconstruction by manually annotated local dendrites for each neuron within image blocks of 512×512×256 voxels (approximately 236×236×512 μm^3). A total number of 1754 successfully reconstructed local morphologies and their corresponding manual reconstructions were used for comparison. **B**. Distribution of the Topological Morphology Descriptor (TMD) distances between the automatic reconstructed morphologies and their corresponding manually annotated dendrites. As a reference, we calculated the average TMD distance of 1000 pairs of manually annotated GPe-projecting CP neurons and indicated by the vertical orange line. **C**. Hierarchical clustering performed for the 121 regions that contained at least 10 neurons. The regions containing less than 10 neurons (somas) were discarded, and all other neurons in the SEU-D15K were used in calculating the regional features, which involved three brain areas consisting of functionally related region sets defined in CCFv3, namely cerebellum (CB), cerebral nuclei (CNU), and cortex (CTX). The regions and brain areas were estimated based on soma location after registration to the CCFv3 atlas. The corresponding brain area for each region was listed at the top of the heatmap. The regional features were represented by the median features of all neuronal 19-dimensional L-Measure^{Vaa3D} features in the corresponding regions. The values of each feature were Z-score normalized separately. The neuronal features exhibited a notable aggregation according to brain areas in general. Source data are provided as a Source Data file.

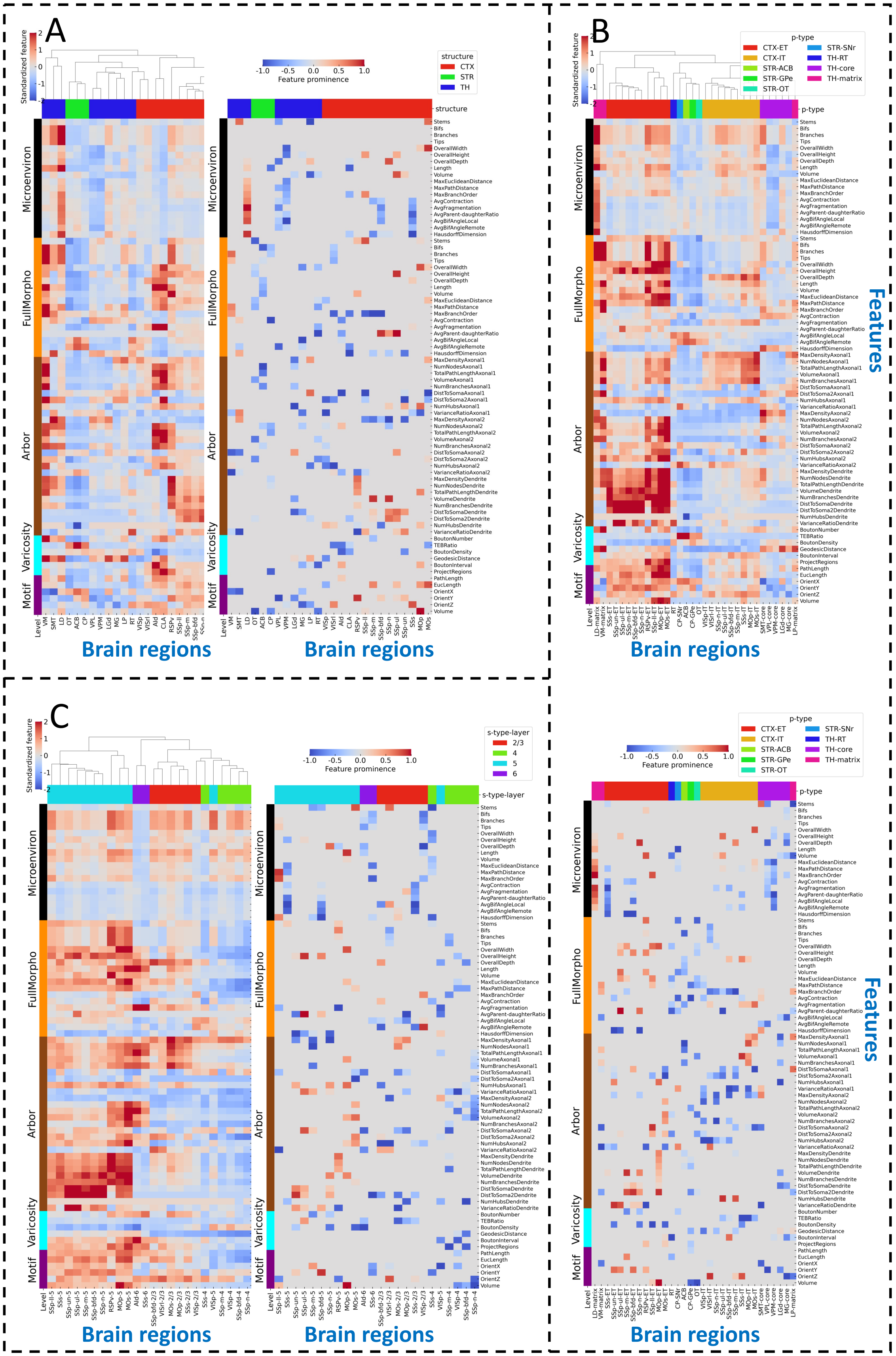
A
B
C
Standardized feature
Feature prominence
structure
CTX
STR
TH
p-type
CTX-ET
CTX-IT
STR-ACB
STR-GPe
STR-OT
STR-SNr
TH-RT
TH-core
TH-matrix
s-type-layer
2/3
4
5
6
Microenviron
FullMorpho
Arbor
Varicosity
Motif
Brain regions
Features

21

Supplementary Figure S2. **Cross-scale feature maps of whole-brain neuron types and subtypes. A**. Left, cross-scale feature map for soma types (s-types) that incorporates five different scales: microenvironment, full morphology, arbor, varicosity, and motif. By combining these features, a set of cross-scale features is obtained. The values of each feature are Z-score normalized by subtracting their mean value and then dividing by their standard deviation. The right and left y-ticks of the map are the feature names and their corresponding morphometry levels, respectively. Hierarchical clustering is applied to all s-types, and the resulting dendrogram is displayed at the top of the map. The x-ticks are sorted according to the dendrogram. Right, the feature prominence map delineates the ten most discriminating features for each s-type, with the prominence scores determined by the ordering of the absolute feature values, and subsequently max-normalized by dividing 10. The prominence values are colored by the signs of their original features value in the cross-scale feature map, with blue indicating a positive value and red indicating a negative value. **B**-**C** are similar maps for projection subtypes (sp-types) and lamination subtypes (sl-types) of cortical neurons, where ET and IT are the extratelencephalic and intratelencephalic projecting subtypes, and 2/3, 4, 5, 6 are the cortical layers of somas. Source data are provided as a Source Data file.

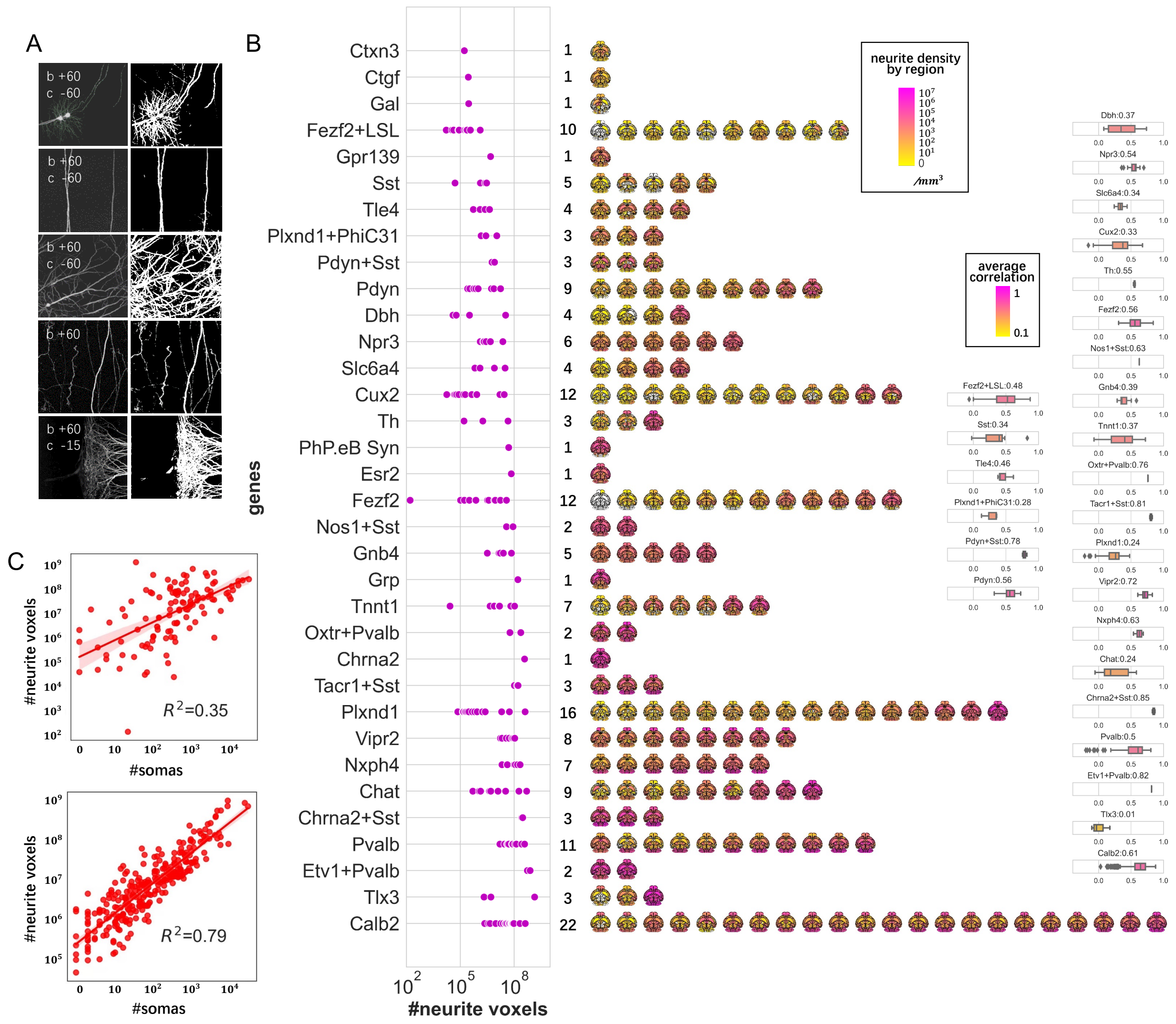

Supplementary Figure S3. **The neurite voxel distributions in fMOST brains. A**. Image blocks exemplifying our neurite segmentation results in fMOST brains. The left and right images are the maximum intensity projections (MIP) of raw images and corresponding neurite segmentation maps. The top left legends on the raw MIPs are the adjustments applied on them for better visualization. "b" and "c" represent brightness and contrast adjustment, while "+" and "-" signify percentage changes for the corresponding adjustments. **B**. Left, distributions of neurite voxel numbers of 183 brains from 34 driver genes. The number of neurite voxels is the total number of voxels identified in a brain image. Each magenta dot represents a brain, and the total number of brains in each line is displayed on the right side. Right, whole-brain neurite patterns. Zoom-out view of whole-brain neurite voxel distribution at a regional level. Box plots on the right side show the correlations between regional neurite distribution patterns of all brain pairs in each transgenic line. **C.** Top, relationship between the number of identified neurite voxels and the total number of annotated somas for each brain. Bottom, relationship between the number of identified neurite numbers and the total number of annotated somas for every region.

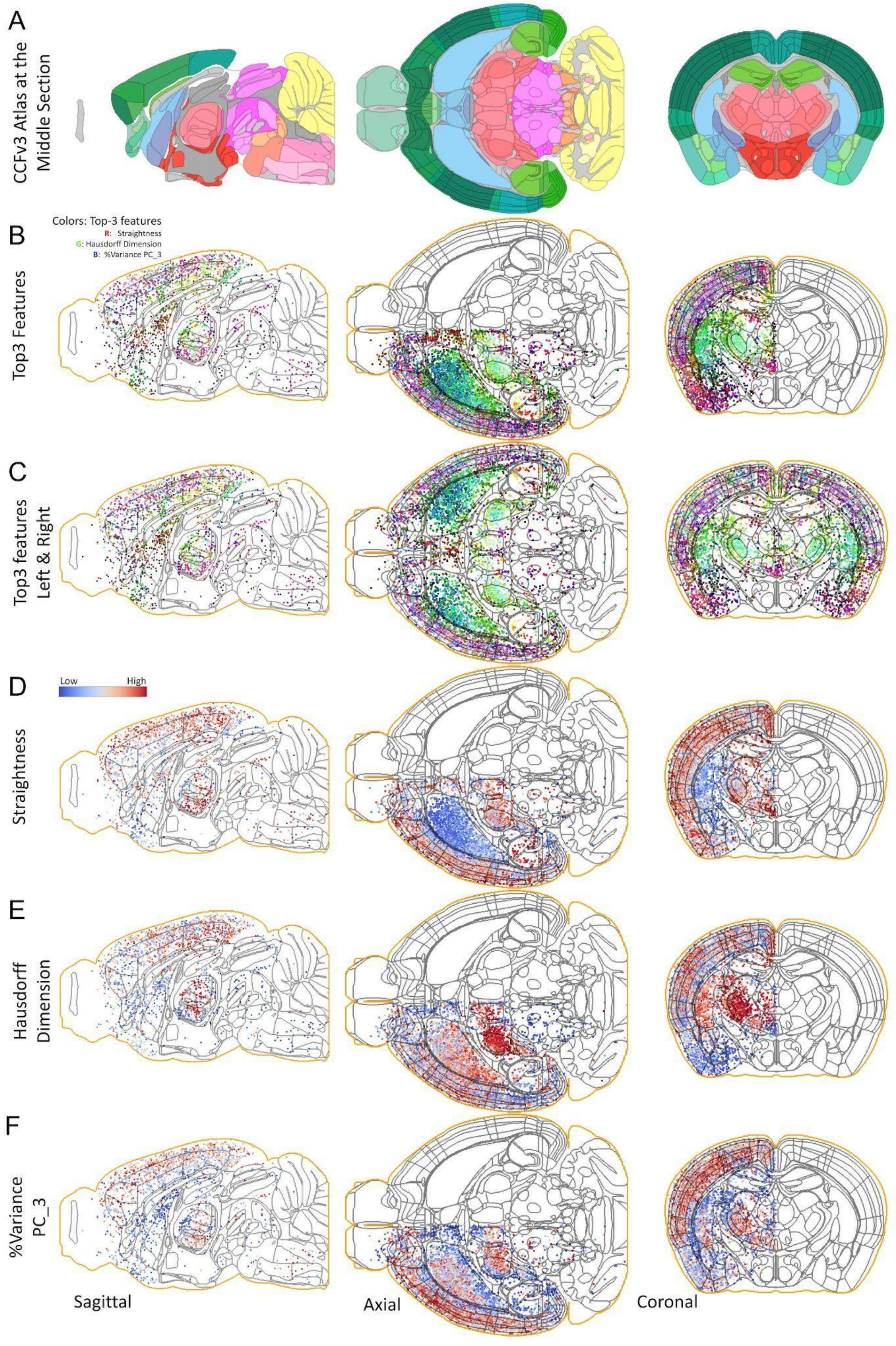
A
CCFv3 Atlas at the Middle Section
B
Colors: Top-3 features
R: Straightness
G: Hausdorff Dimension
B: %Variance PC_3
Top3 Features
C
Top3 features Left & Right
D
Low
High
Straightness
E
Hausdorff Dimension
F
%Variance PC_3
Sagittal
Axial
Coronal

52

Supplementary Figure S4. **Whole-brain microenvironment feature distributions along middle sections of the sagittal, axial, and coronal views. A.** The sagittal, axial, and coronal middle sections of the CCFv3 atlas. Brain areas and regions are colored following the convention of the CCFv3 atlas. Cortical regions are in green-blue colors, cerebral nuclei regions are in cyan, brain stem regions are in red, midbrain regions are in pink, and cerebellar regions are in yellow. The gray lines are the boundaries of CCFv3 regions. **B**. Projection of the top 3 discriminating morphological microenvironment features selected through minimum Redundancy-Maximum Relevance (mRMR) on the middle sections. The top 3 features are: average straightness, Hausdorff Dimension, and variance percentage of the third component of all nodes, and they are encoded in the red (R), green (G), and blue (B) channels of the image. The feature values are normalized and histogram-equalized to the unsigned 8-bit integer range. Only neurons within a 1-millimeter range in both directions are included. The outermost boundary of the CCFv3 brain template is outlined in orange, and the microenvironments on the right hemisphere are flipped to the left hemisphere. **C**. Similar to panel B, but the right hemispheric microenvironments are not flipped. **D-F**, The distributions for the three features are displayed separately at each view.

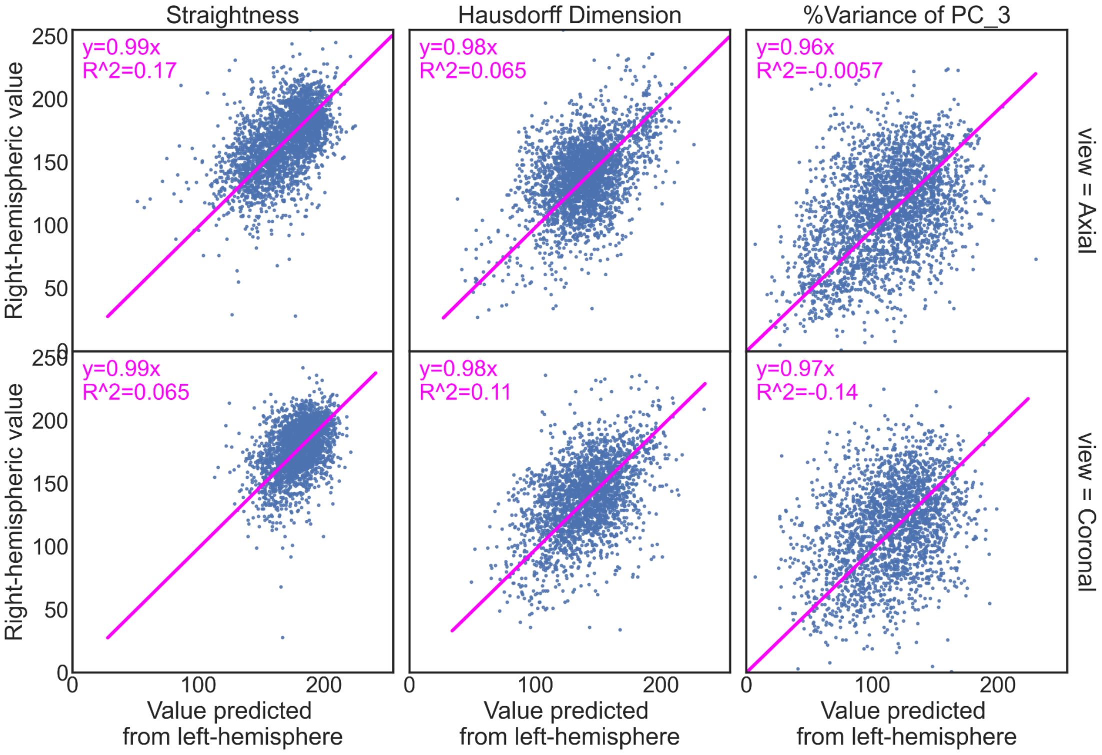

Supplementary Figure S5. **Correlations between feature values in the right hemisphere and those predicted from microenvironments of the left hemisphere on the axial and coronal middle sections.** The values on the y-axis are the feature values of microenvironments in the right hemisphere, while the values on the x-axis are predicted features for mirrored positions through multidimensional linear interpolation using features of the left hemisphere. The points are fitted with the linear function $y = a \cdot x$.

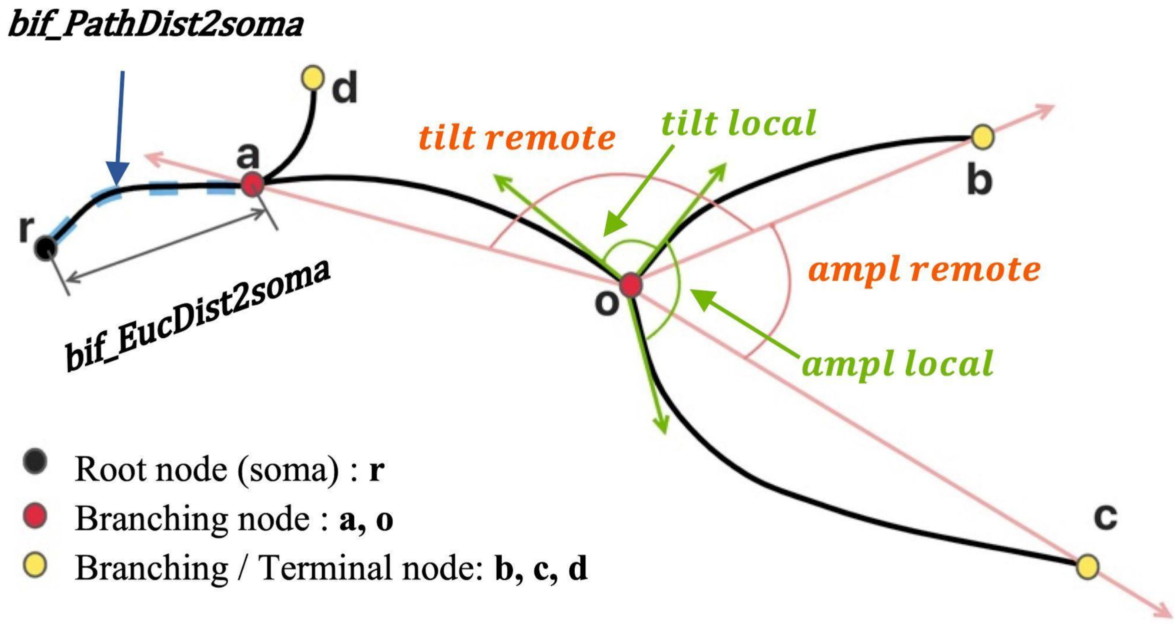

Supplementary Figure S6. **Diagram illustrating the definition of several critical local morphological features leveraged in full morphology analysis.** The features "bif_EucDist2soma" and "bif_PathDist2soma" are Euclidean and path distances from the current bifurcation point to the root node (soma). "tilt remote" is the "bif_tilt_remote" defined in L-Measure^{Vaa3D}, which represents the angle between the parent node, the current bifurcation point, and one of its two daughter critical nodes. The smaller angle of the two angles formed with the two daughter nodes is used. A critical node here is a topological critical point that is either a terminating point, a bifurcation point, or a root point. The feature "tilt local" is similar to "tilt remote" except the anchor points are not critical points, but instead are the nearest compartments along the branches. The features "ampl remote" and "ampl local" are similar to "tilt remote" and "tilt local" except that the angle is formed by daughter points and the current branching point.

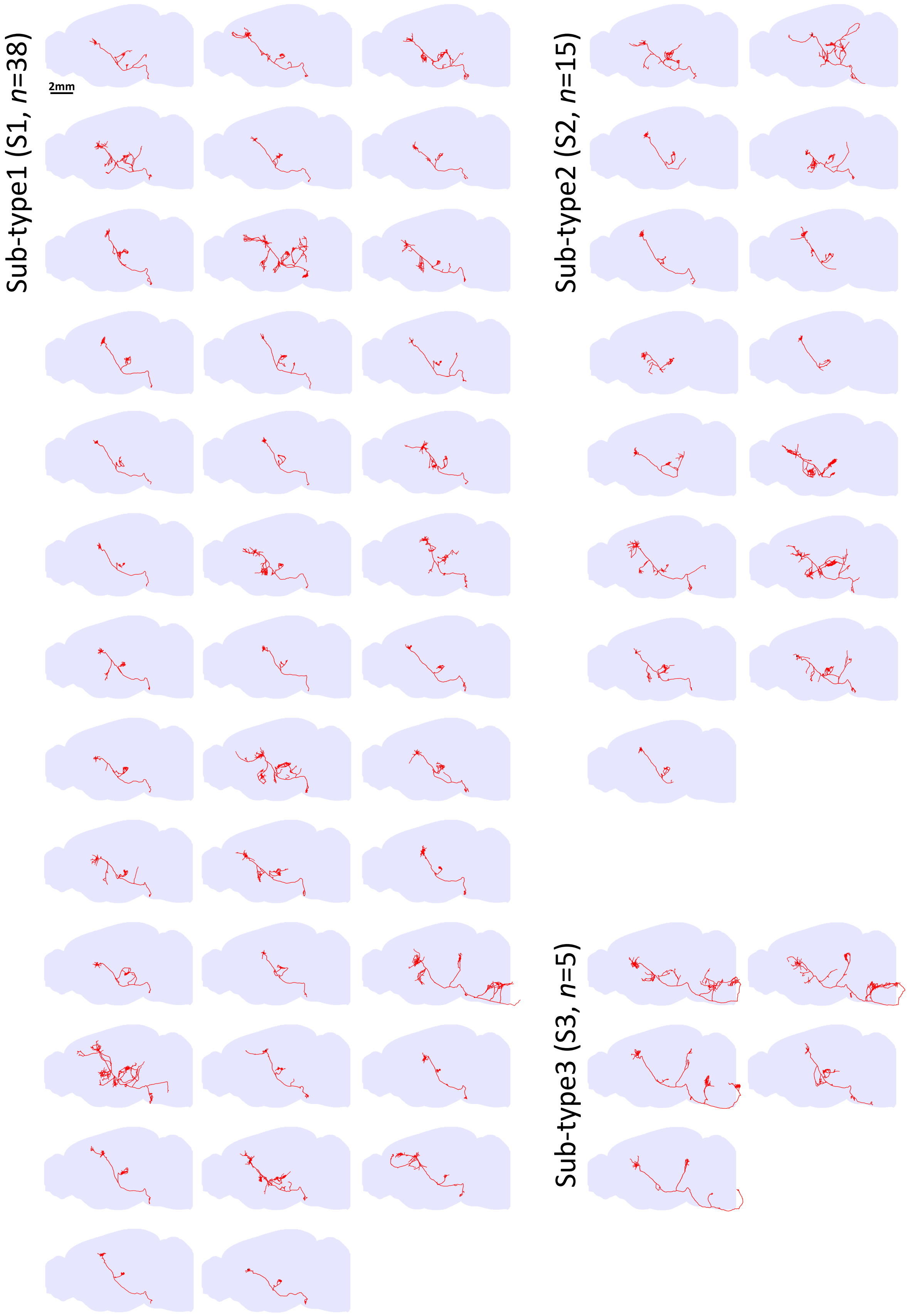

87

Supplementary Figure S7. **Sagittal projections of the three subtypes of L5 ET-projecting SSp-m neurons in the cortex.** L5 ET-projecting SSp-m neuron is a fine-grain extratelencephalic projecting cortical neuron type SSp-m with the soma located at cortical layer 5 (L5). All 38 subtype-1, 15 subtype-2, and 5 subtype-3 neurons are overlaid on the sagittal view of the CCFv3 template. The three subtypes are classified based on the terminal coordinates of their primary tracts using K-Means clustering.

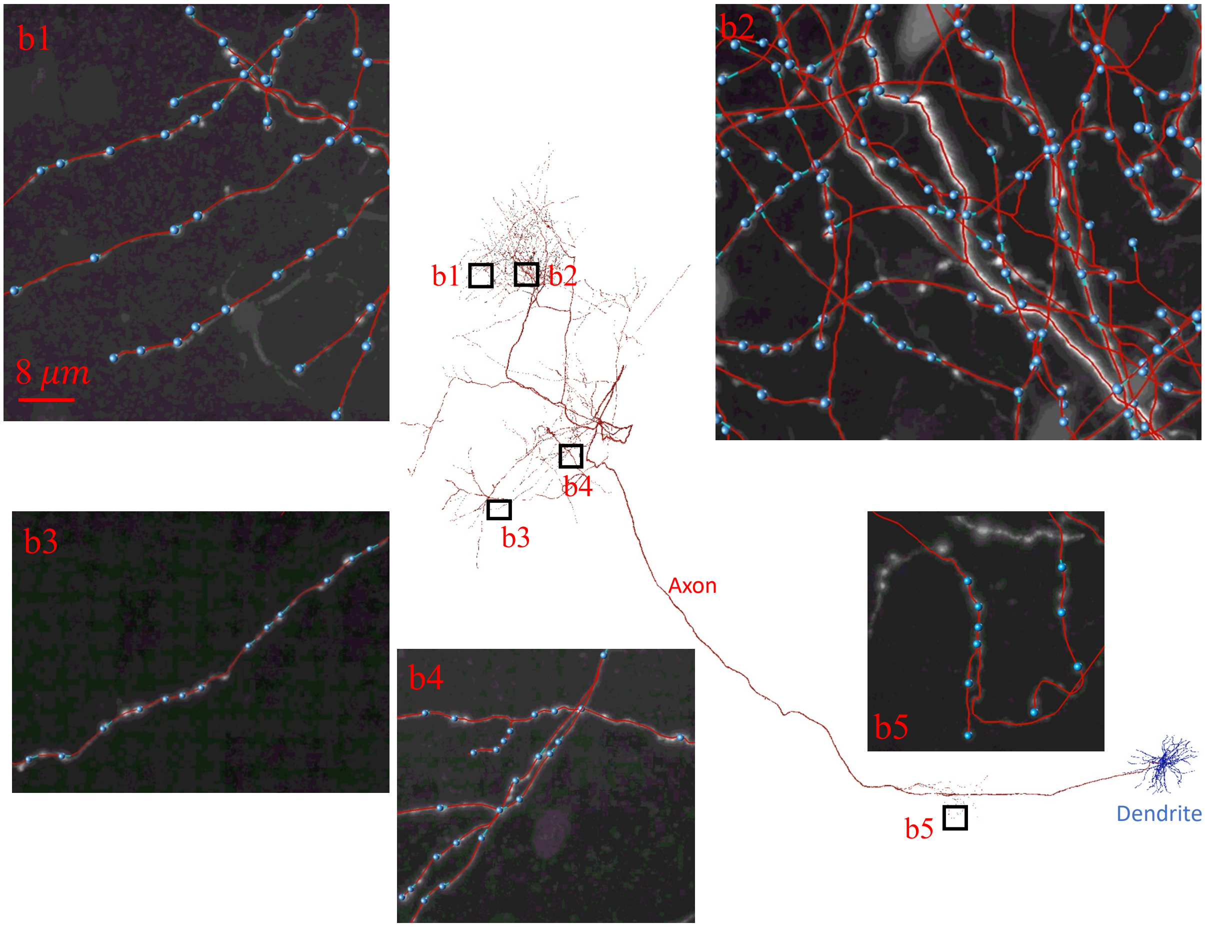

Supplementary Figure S8. **A thalamic VPM neuron with detected** varicosities **overlaid.** Five zoom-in blocks, b1-5, are displayed through maximum intensity projection (MIP), and the reconstructed skeletons are overlaid in place with the image. The cyan dots are the detected varicosities. The full morphology of the neuron is illustrated in the middle of these blocks, with dendrites colored in blue and axons in red.

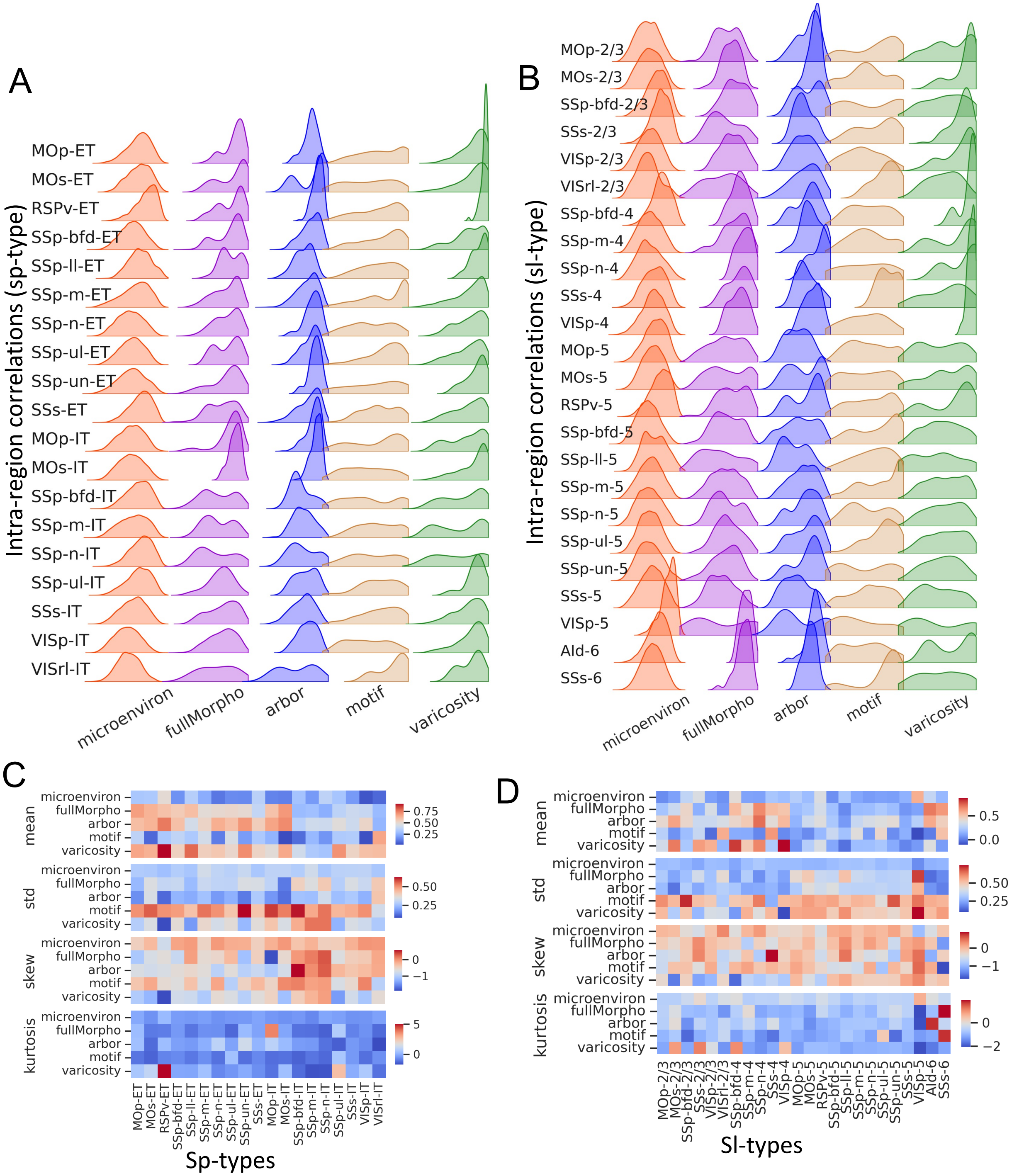

Supplementary Figure S9. Intra-region correlations for projection subtypes (sp-types) and lamination differentiated subtypes (sl-types) of cortical neurons. **A** and **B**. Density plots of the intra-region correlation distributions for sp-types and sl-types at different morphometry levels. **C** and **D** Heatmap of the first (mean), second (std), third (skew), and fourth (kurtosis)-order statistics of intra-regional correlation distributions for sp-types and sl-types respectively.

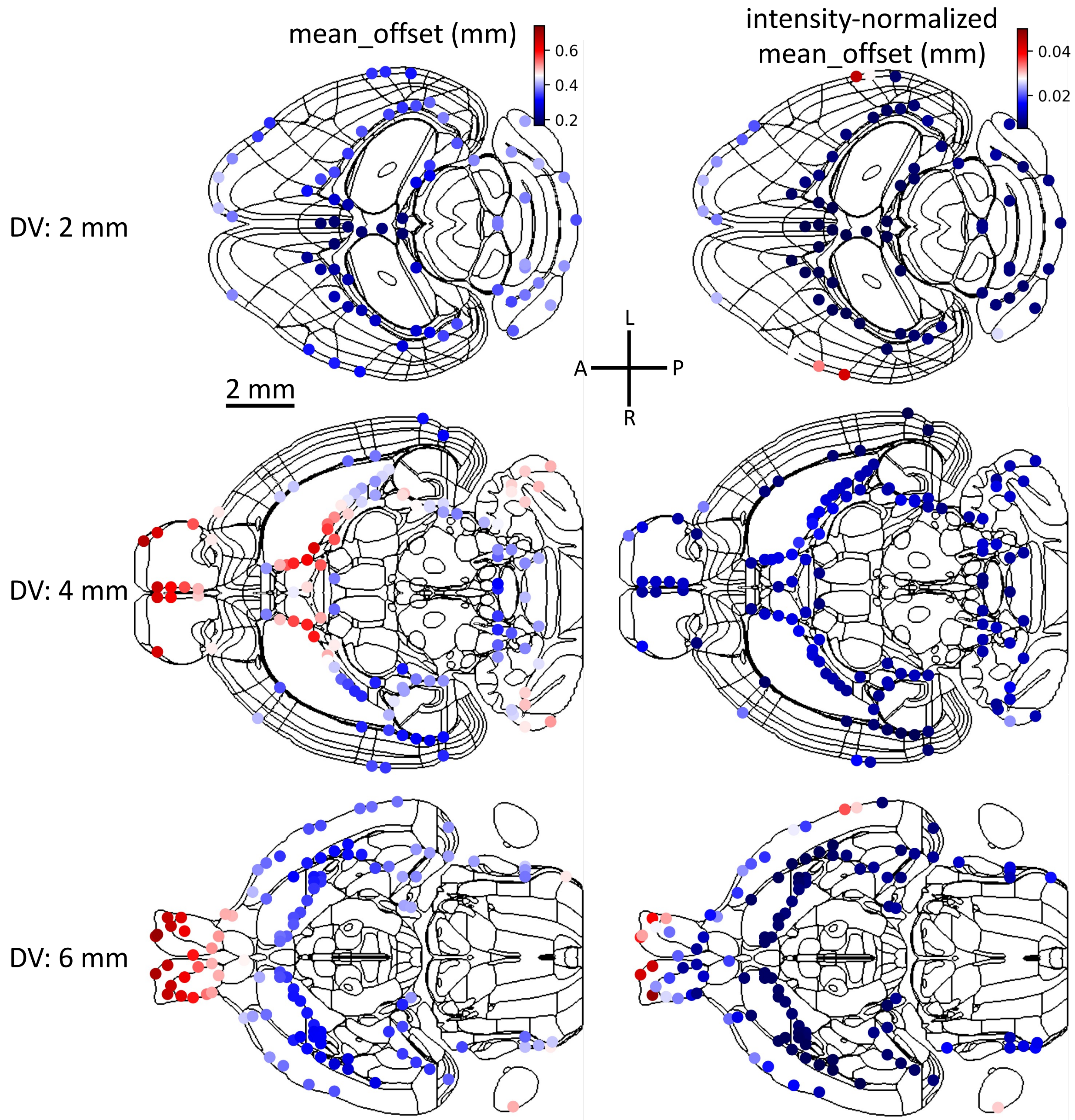

Supplementary Figure S10. **Registration robustness on landmarks.** Three horizontal sections of CCFv3 atlas with mean offsets (left) and intensity-normalized mean offsets (right) of landmarks overlaid. The mean offset for each landmark was the average offset of that landmark point on all brains analyzed. The intensity-normalized mean offset was calculated through dividing the mean offset by the standard deviation of intensities of the mapped landmarks on subject brains. To simplify the representation, three horizontal sections along the dorsal-ventral (DV) axis were displayed, corresponding to 2, 4, and 6 millimeters from the original point of CCFv3 atlas. Landmarks within 0.25 millimeters of each section were mapped to that section. Landmarks were color-coded according to their offset values, with separate representations for mean offset and normalized offset. The black outlines in each section indicate the boundary outlines of the brain regions. Scale bar: 2 mm.

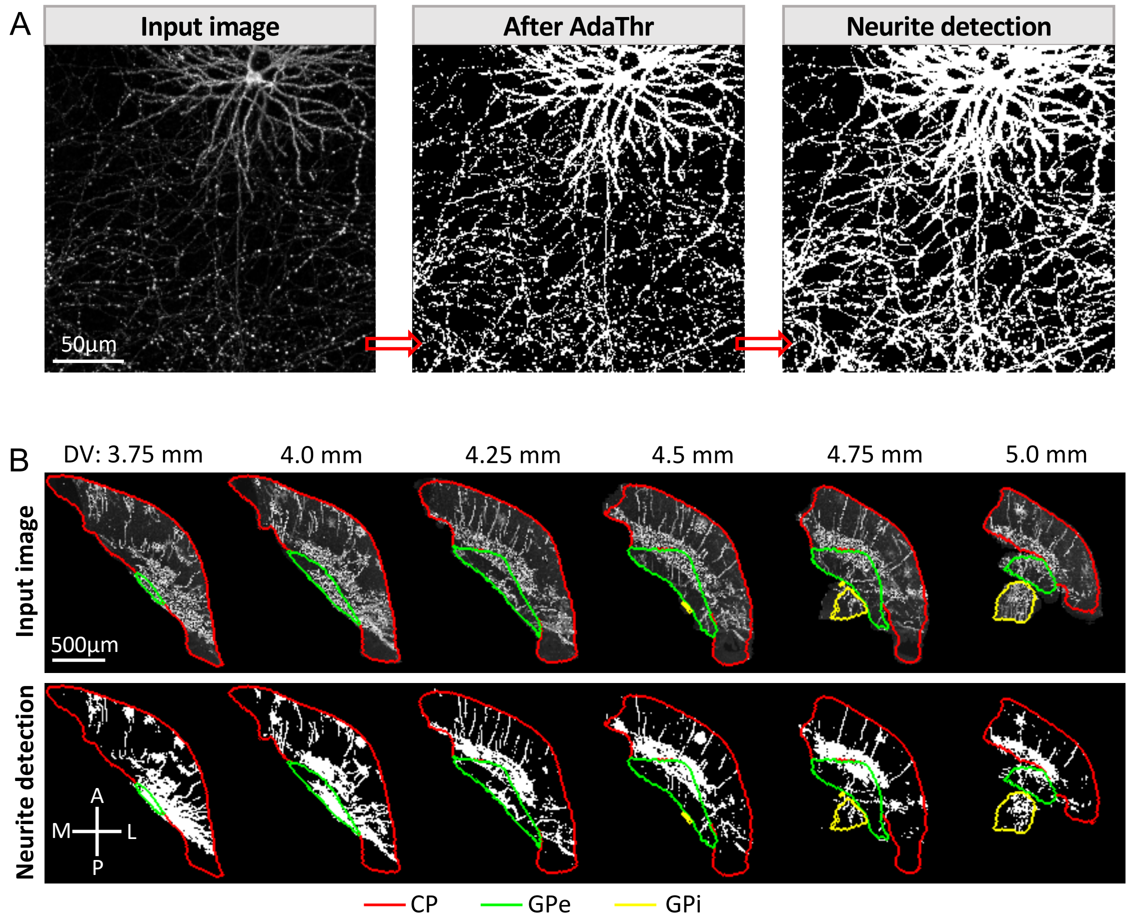

Supplementary Figure S11. **Neurite detection examples. A.** Illustration of neurite detection for a neuronal image block with dendrites and local axons. The first, second, and last columns are the input image, intermediate results after adaptive thresholding filter, and the final neurite detection. Scale bar: 50 µm. **B**. The input neuronal images and corresponding detections for the CP (red), GPe (green), and GPi (yellow) regions. We displayed six sections along the dorsal-ventral (DV) axis, specifically at 3.75 mm, 4.0 mm, 4.25 mm, 4.5 mm, 4.75 mm, and 5.0 mm from the origin point at the dorsal side of the atlas of a fMOST brain. The atlas is reverse-mapped from the CCFv3 atlas based on the registration matrix. Only the regions within the three brain regions (CP, GPe, GPi) are included for visualization. Scale bar: 500 µm.

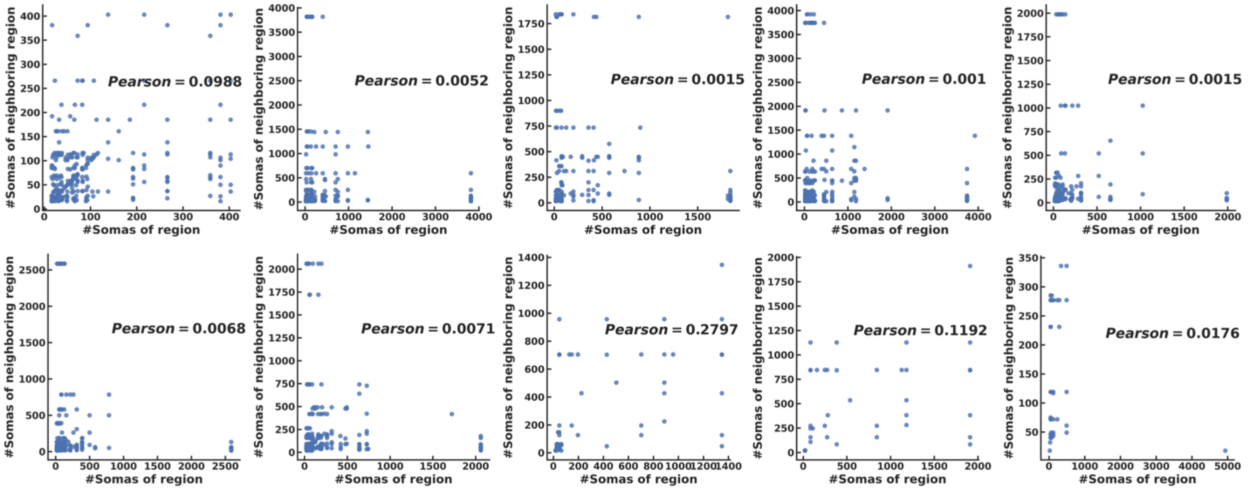

Supplementary Figure S12. **Relationship between the numbers of annotated somas in neighboring regions.** Scatter plots of the numbers of somas in neighboring regions for the ten brains with the highest numbers of total annotated somas. Each point represents the number of somas annotated in a pair of regions. A region pair is defined as two CCFv3 regions with a minimum distance of less than 125 μm. Only regions containing more than 15 somas are considered for the sake of statistical reliability. The inset legend in each panel is the Pearson correlation coefficient of fitted line (not shown).

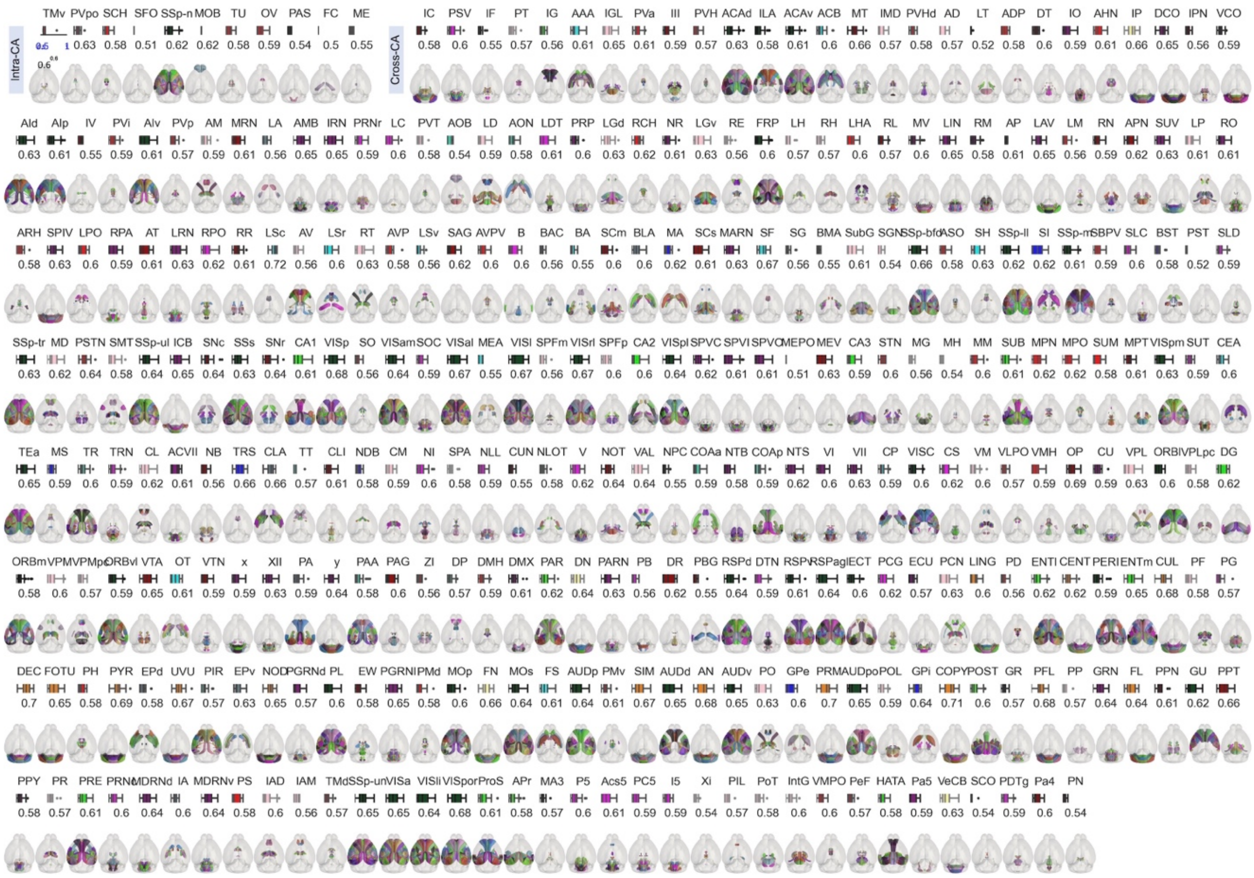

Supplementary Figure S13. **Correlated region sets for 313 brain regions.** Horizontal projections on the CCFv3 template of regions with a Spearman correlation coefficient of at least 0.5 with the target region (specified at the top of each brain image). The box plot on the top of each brain image is the distribution of the pairwise correlations between these regions and the target region, with the box colored by compound areas (CA) as in **Figure 2A**. An intra-CA region set contains only regions from the same compound area, while cross-CA set contains regions from at least two different compound areas.

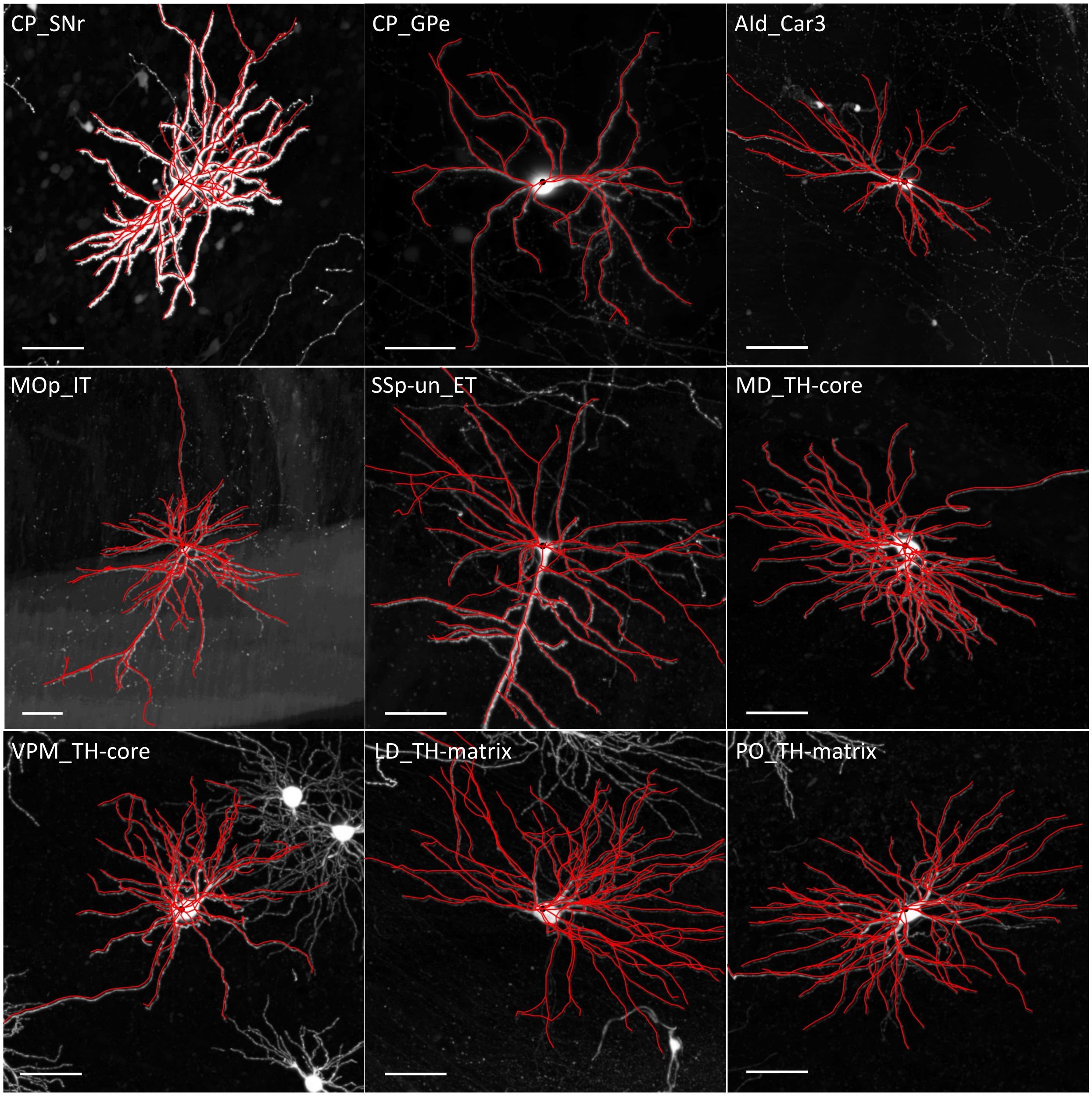

Supplementary Figure S14. **Examples of automatically reconstructed local dendrites.** Nine image blocks from different neuron types (or subtypes) are presented. Each neuron is named by concatenating its region name where the soma is located and its projection type, separated by an underscore. For instance, CP_SNr and CP_GPe refer to SNr and GPe-projecting CP neurons, while AId_Car3 designates claustrum-like AId neurons. Additionally, MOp_IT and SSp-un_ET represent intratelencephalic MOp neurons and extratelencephalic SSp-un neurons, respectively. TH-core and TH-matrix refer to the core and matrix projection types. The morphologies are displayed in dots and lines, with black dots indicating somas, and red lines representing the fibers. To enhance visualization, the morphologies were shifted by 2 voxels horizontally and vertically. Scale bar: 20 μm.

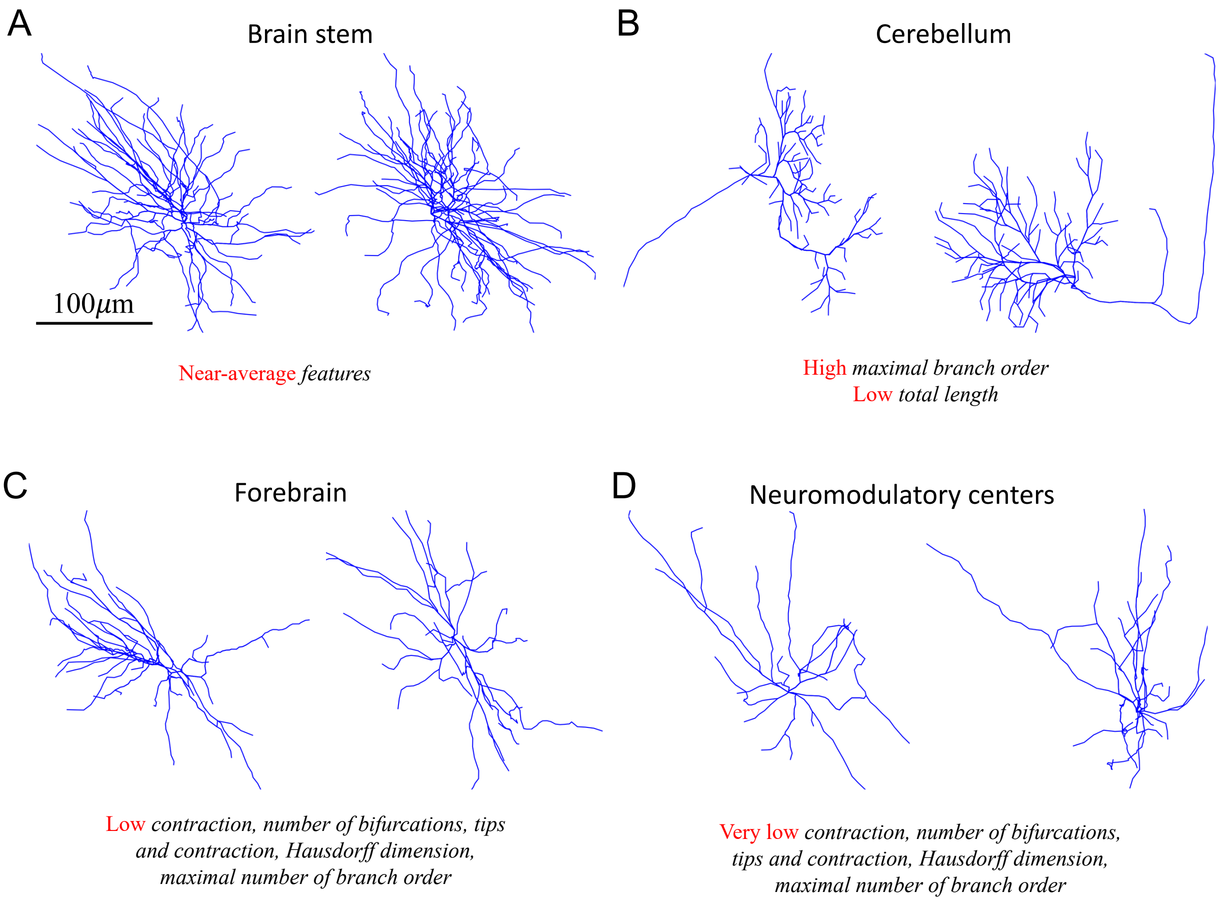

***Supplementary Figure S15.* Representative neurons for brain stem, cerebellum, forebrain and neuromodulatory centers.** Sagittal views of the local morphologies of two representative neurons for each brain area are displayed. The most discriminative L-Measure^{Vaa3D} features for each area are summarized below the examples. Scale bar: 100 μm.

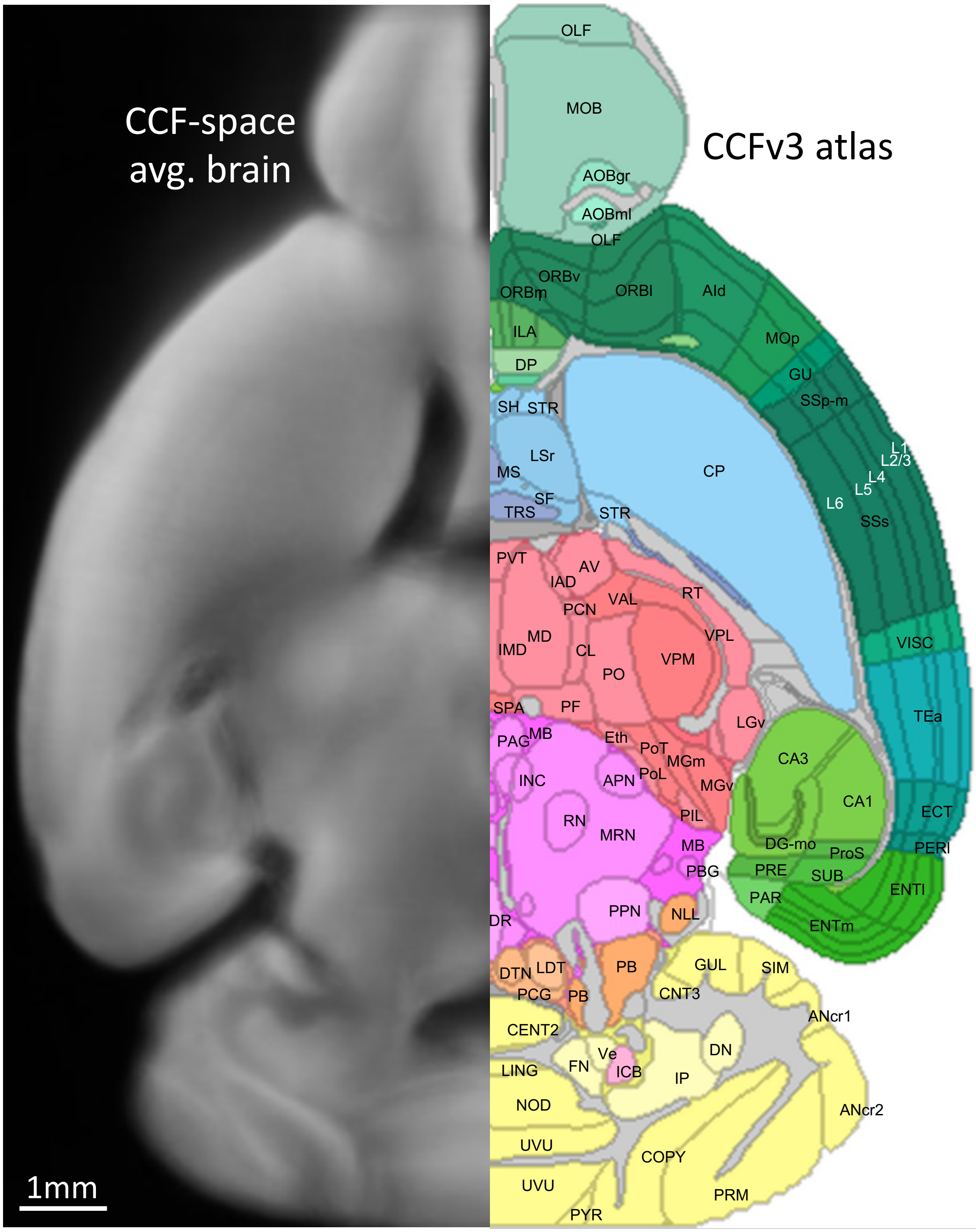

Supplementary Figure S16. **The CCF-space average brain.** Left, the middle axial section of the average brain generated by averaging 191 whole brain images analyzed in this work. Right, the corresponding axial section of CCFv3 atlas with region names explicitly labeled.

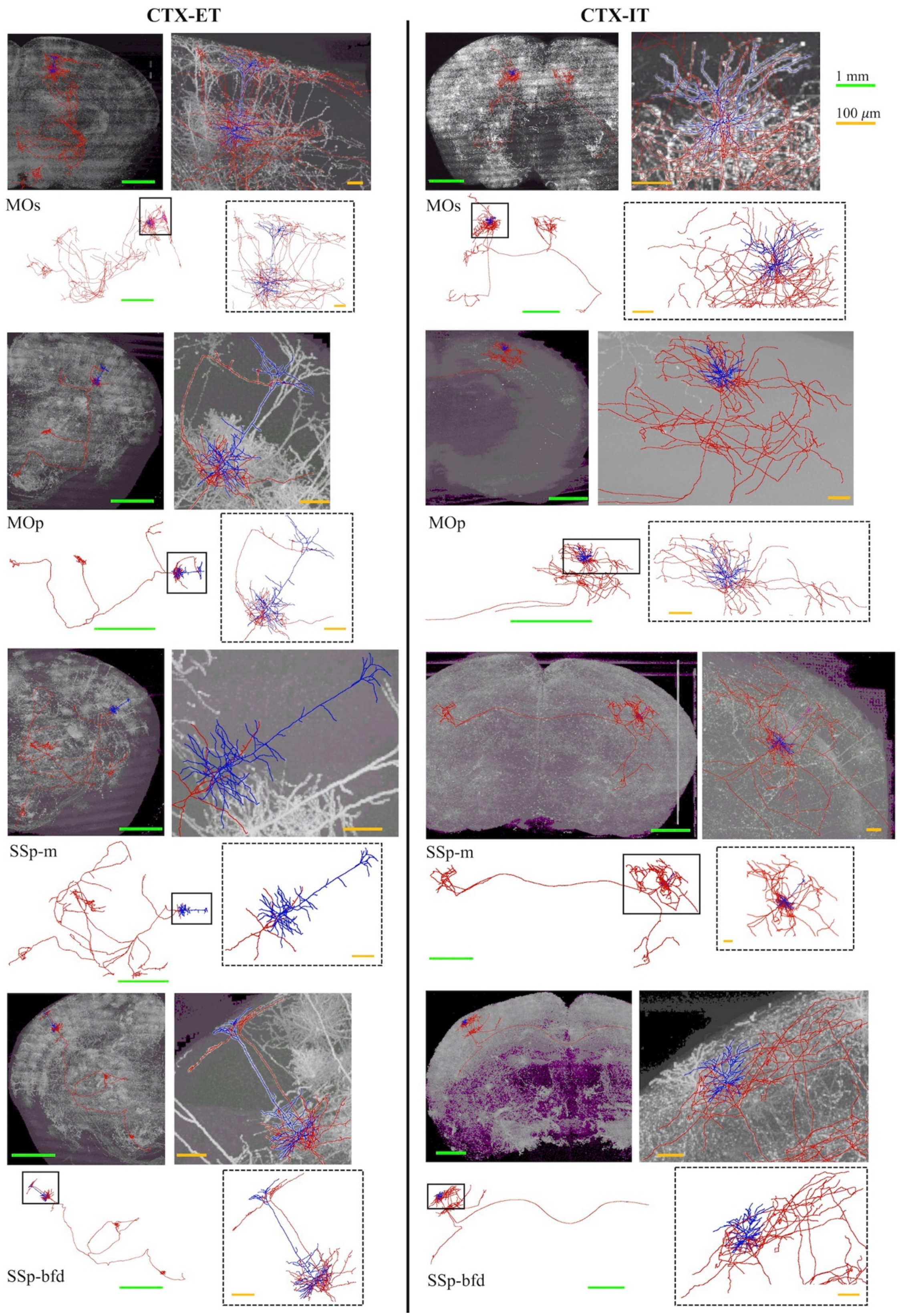

Supplementary Figure S17. **Dendritic arbors of cortical neurons.** Coronal views of dendritic arbors of the ET-projecting (left) and IT-projecting (right) subtypes of four cortical neuron types (MOs, MOp, SSp-m, SSp-bfd). For

each neuron: top left panel, coronal view of single neuron morphology overlaid on brain image; bottom left, the single neuron morphology, with dendrites highlighted by a solid rectangle; top right, dendrites overlaid on the image; bottom right, dendrites. The morphology is color-coded by the neurite types, with dendrites in blue, and axons in red. Scale bars: green, 1 μm; yellow, 100 μm.

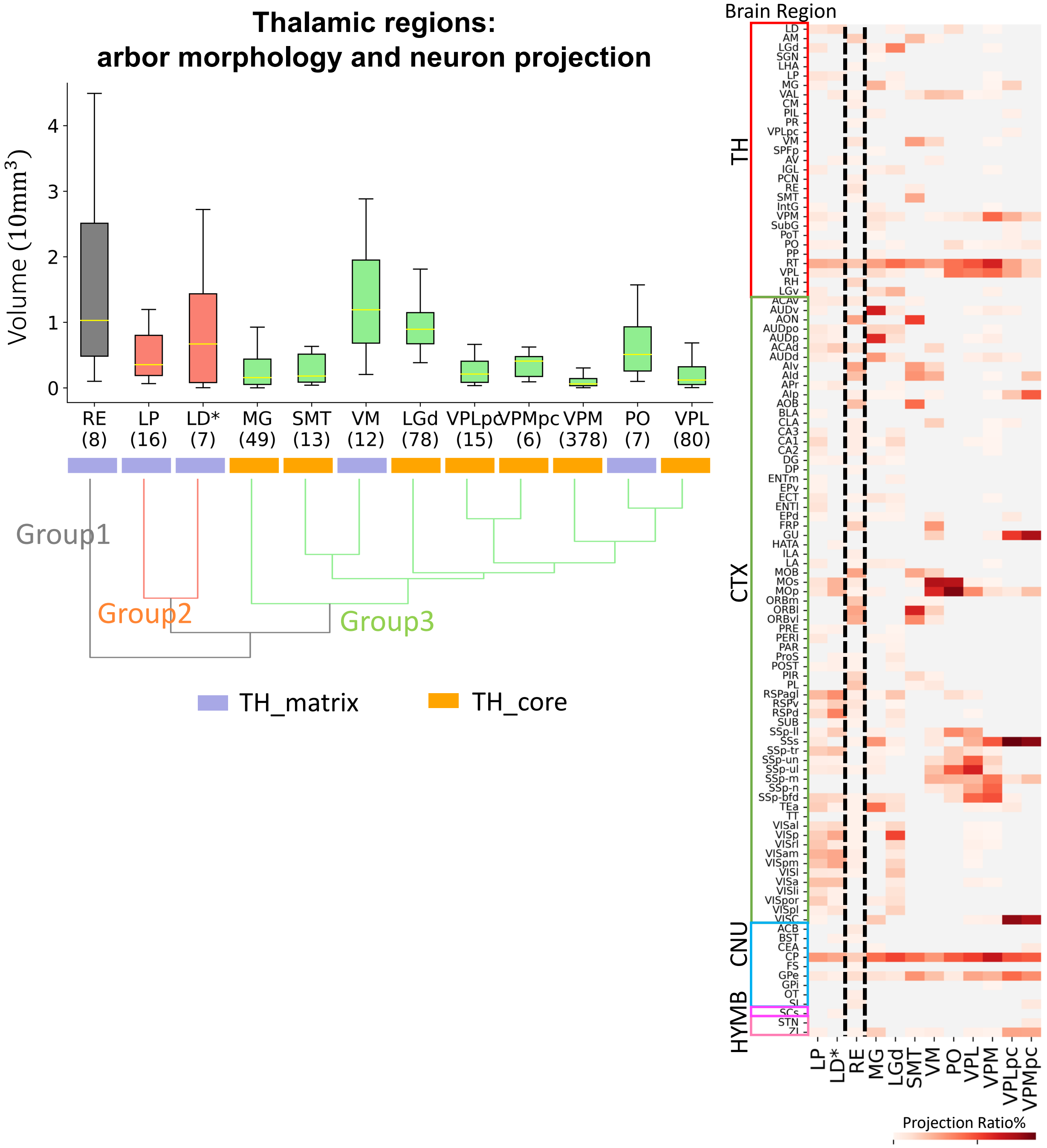

Supplementary Figure S18. **Diversity and stereotypy of arbors of thalamic neurons.** Left, box plot showing the arbor volume of 12 thalamic neuron types. The dendrogram shows groups obtained by hierarchical agglomerative clustering based on the combination of 8 morphological features (mean and standard deviation of "#branch", "volume", "max_density", "dist2soma") and their projection strength vector across the brain regions. Right, heatmap of the whole-brain projection strength distributions for the 12 types. Each row is a projection region, grouped by their brain areas, which are highlighted at the left of the heatmap. Each row is an s-type region for the analyzed neuron sorted according to the clustering results of the left panel. Given that the "thalamic core" LD neurons only has 3 neurons, the projection class "thalamic matrix" of LD neurons (LD*) is displayed.

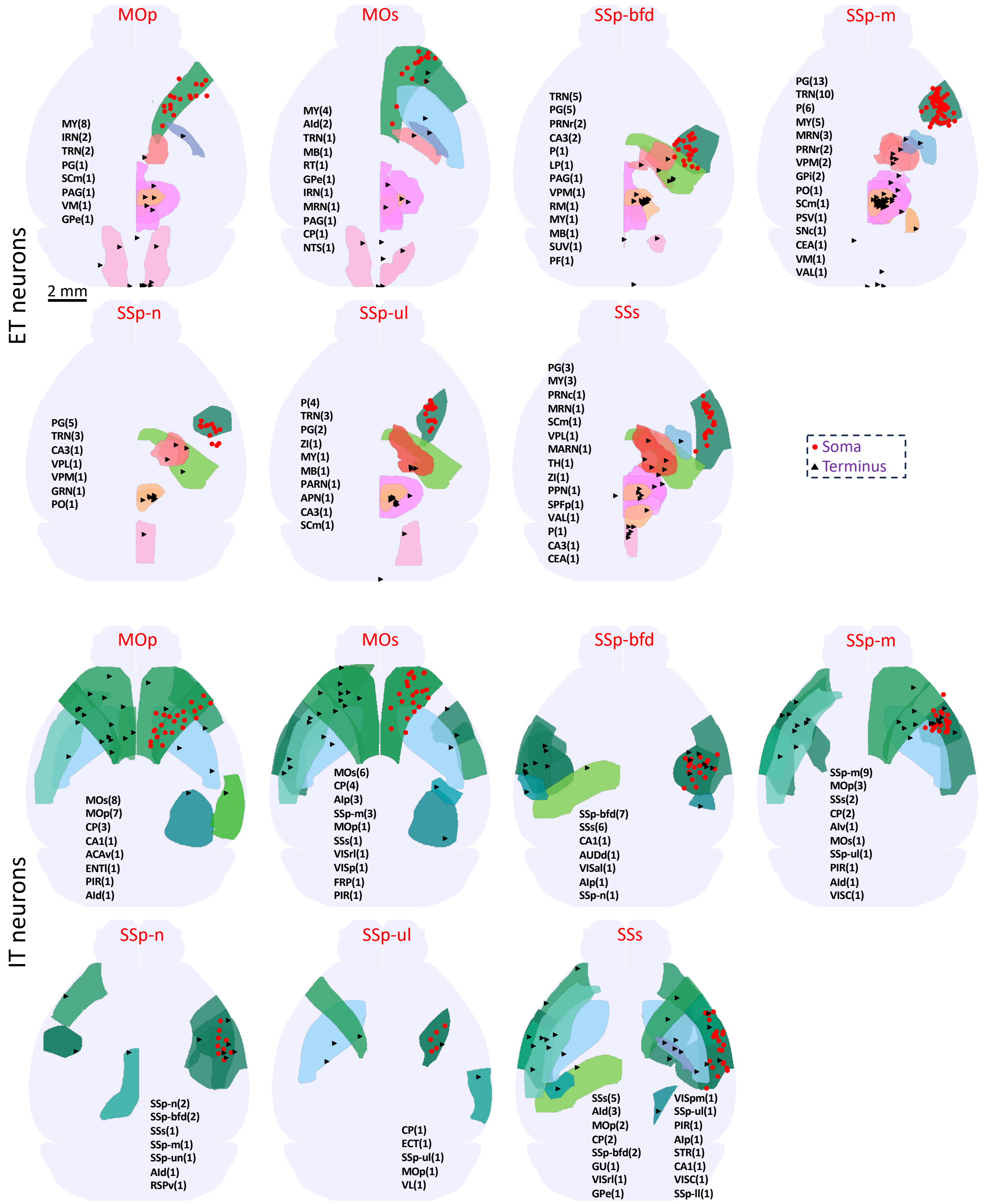

Supplementary Figure S19. **Projection topographical organizations of the ET and IT projecting neurons of 7 cortical types.** Horizontal views of somas (red dots) and the terminal points of primary axonal tracts (black triangles) of neurons belonging to the same projection subtypes are mapped onto the standardized CCFv3 template (ghost white). Regions where somas and terminal points located are highlighted with an alpha value of 0.5, color-coded according

to the CCFv3 atlas. The names of these regions are explicitly provided on the respective brains, with source regions in red and terminal regions in black. The number of points within each region is specified in parentheses after the region name. Large brain areas, such as medulla (MY), midbrain (MB), pons (P), thalamus (TH), striatum (STR), and lateral ventricle (VL), are not shown to avoid obscuring other regions. Scale bar: 5 mm.

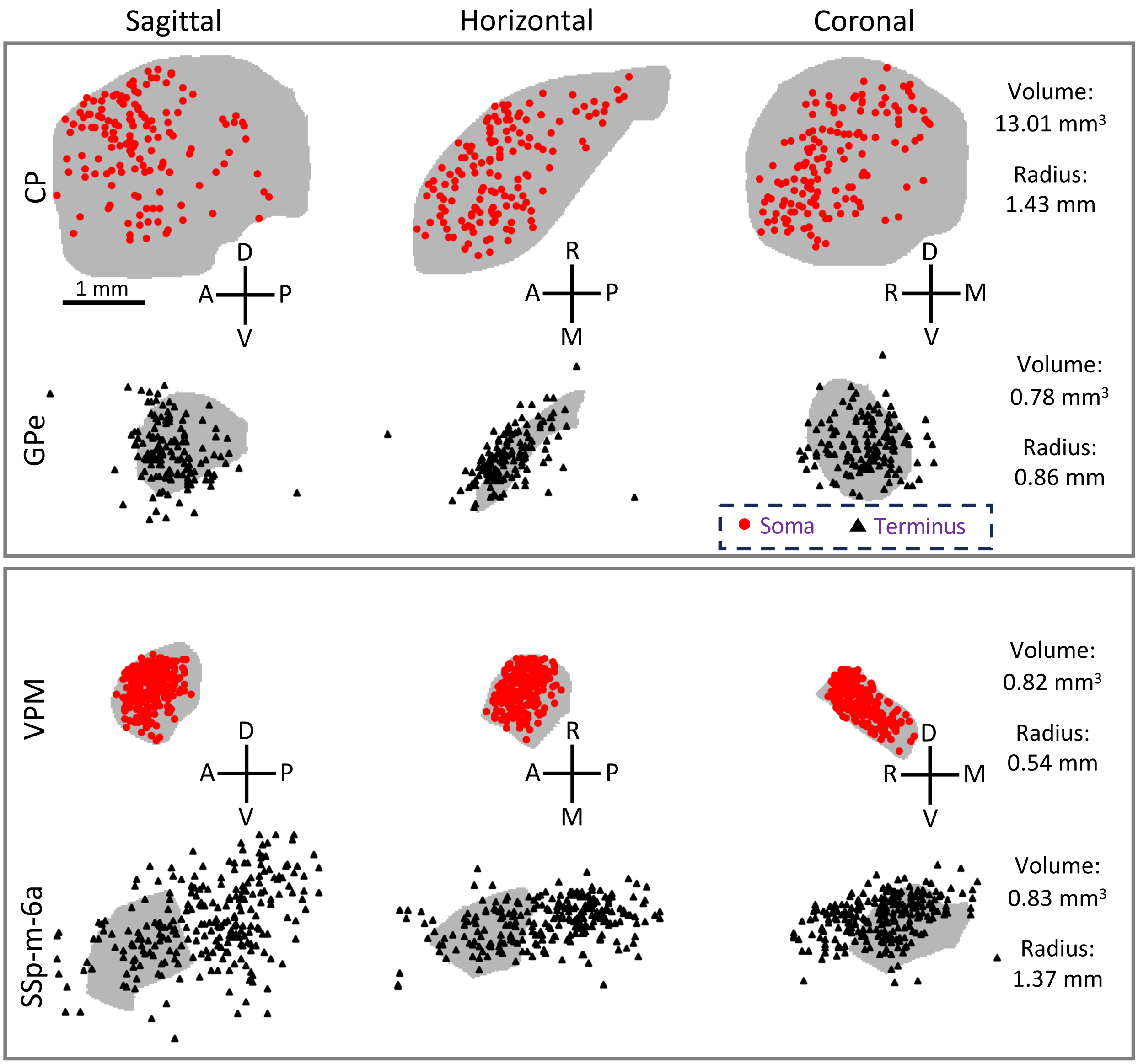

Supplementary Figure S20. **Projection topography for VPM neurons and GPe-projecting CP neuron.** The primary sourcing and terminating regions are indicated with light gray mask. Somas and primary axonal tract termini are represented by red dots and black triangles, respectively. For each region, sagittal, horizontal, and coronal views are provided. Only the regions with the highest number of termini are included. Volumes of regions and radii of somas or termini are displayed on the right side. Details on radius calculation can be found in the **Methods** section. Scale bar: 1 mm.

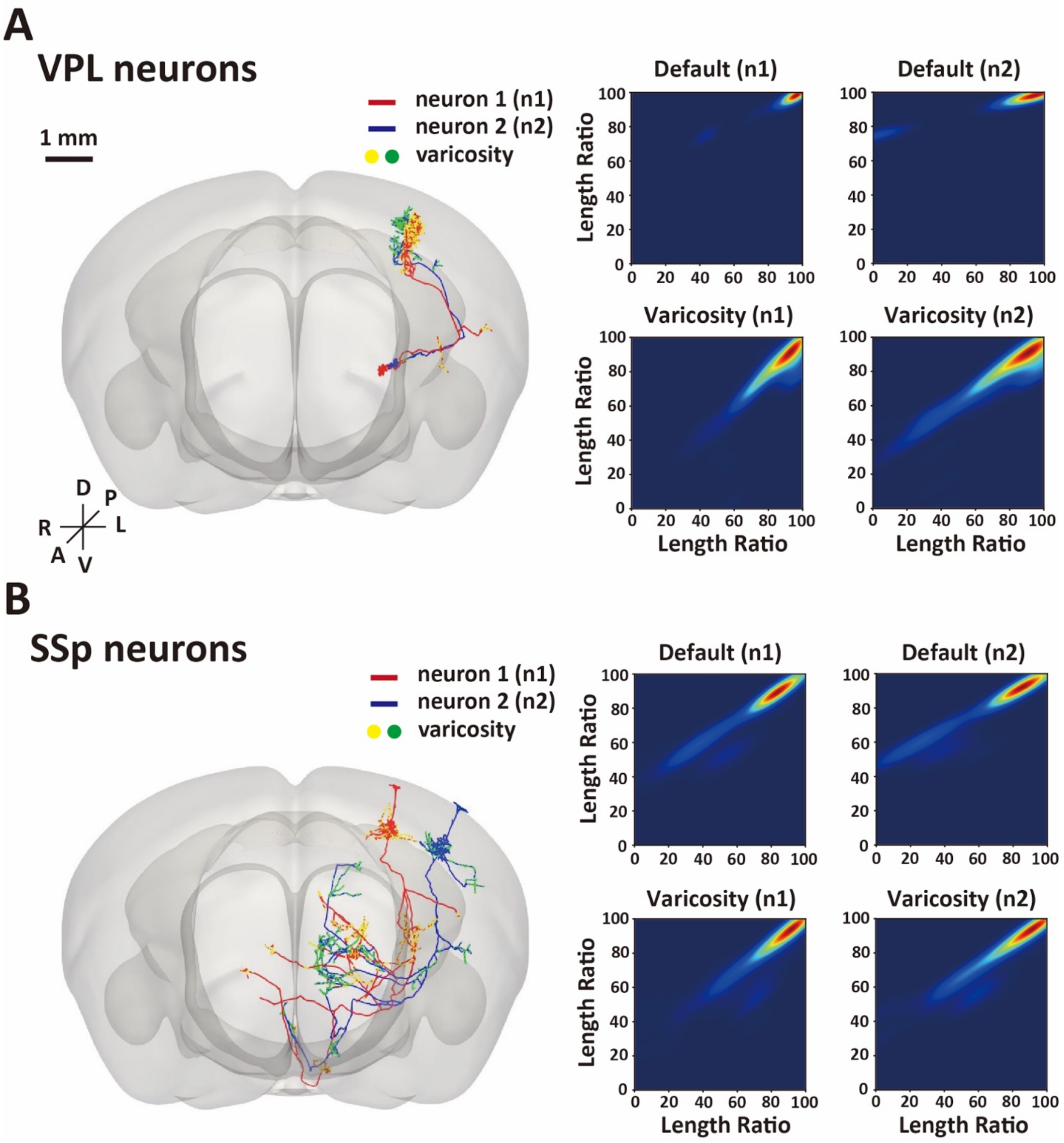

***Supplementary Figure S21.* Similar varicosity distributions in independent but morphologically similar neurons.** **A.** Left, coronal view displaying the morphologies of two VPL neurons (neuron 1, n1, in red; neuron 2, n2, in blue) overlaid on the CCFv3 template. Yellow and green dots represent varicosities of neuron 1 and neuron 2, respectively. Right, heatmap depicting Topological Morphology Descriptor (TMD) persistent lengths for axons ("Default") and varicosities ("Varicosity") of the neurons. The lengths represent Euclidean distances between somas and starting points (topologically near the soma, X-axis) or terminal points (topologically far from the soma, Y-axis). Subsequently, these lengths are normalized by the maximum length to create percentiles, referred to as "Length Ratio". **B.** Comparable components to **A**, but focusing on two morphologically similar neurons from the Primary Somatosensory area (SSp). Scale bar: 1 mm.

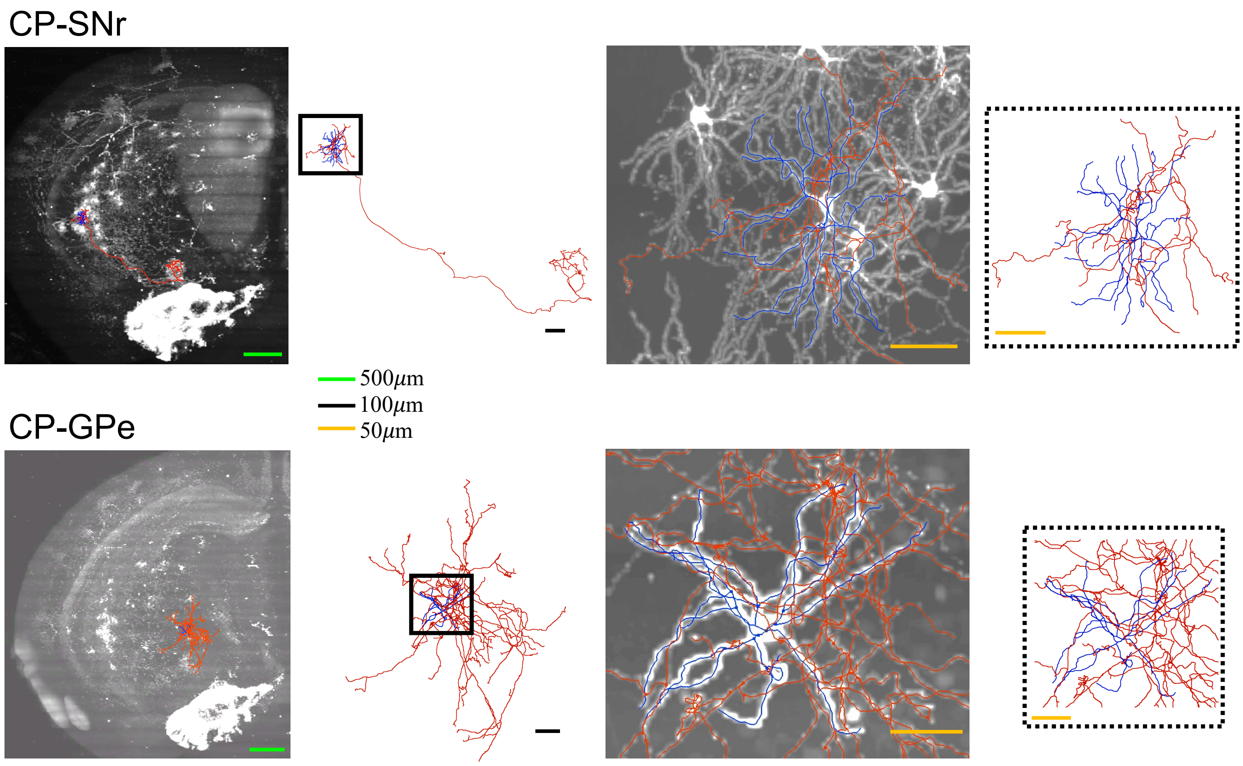

Supplementary Figure S22. **Local axons of CP neurons.** Top panels: coronal views of a SNr-projecting CP neuron; Bottom panels: coronal views of a GPe-projecting CP neuron. From left to right, single neuron morphologies overlaid in the whole brain images, single neuron morphologies with neurites highlighted by black rectangles, zoom-in views of local morphologies overlaid on images, zoom-in views of local morphologies. Neurites are color-coded, with dendrites in blue, and axons in red. Scale bars: green, 500 μm; black, 100 μm; yellow, 50 μm.

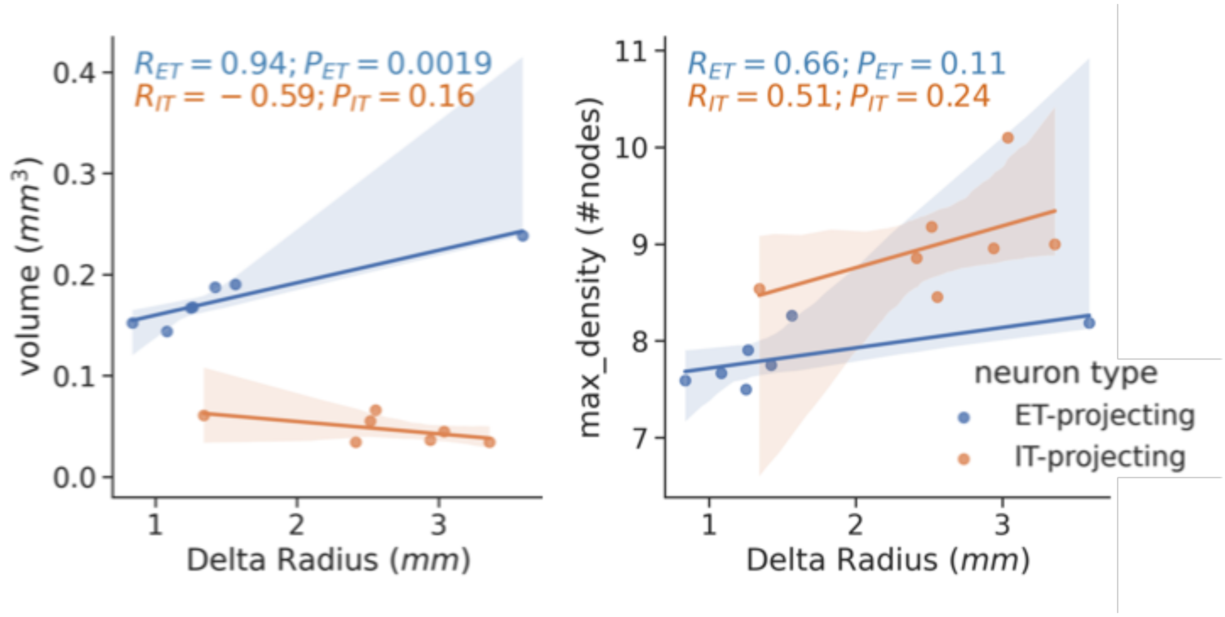

Supplementary Figure S23. **Cross-scale diversity between dendritic arbors and projection patterns.** The projection patterns were estimated using the Delta Radius, calculated as the radius of primary axonal tract termini minus the radius of the somas for neurons from a given subtype. The feature "volume" represents the total volume of the dendritic arbor. The feature "max_density" represents the highest density observed among all compartments, with density defined as the count of compartments within a specified neighborhood. Blue dots and orange-yellow dots represent extratelencephalic (ET) projecting and intratelencephalic (IT) projecting cortical neurons, respectively. The "R" and "P" values stand for the Pearson correlation coefficient and *p*-value, respectively, obtained from the linear fitting statistics for the respective types of projections. Source data are provided as a Source Data file.

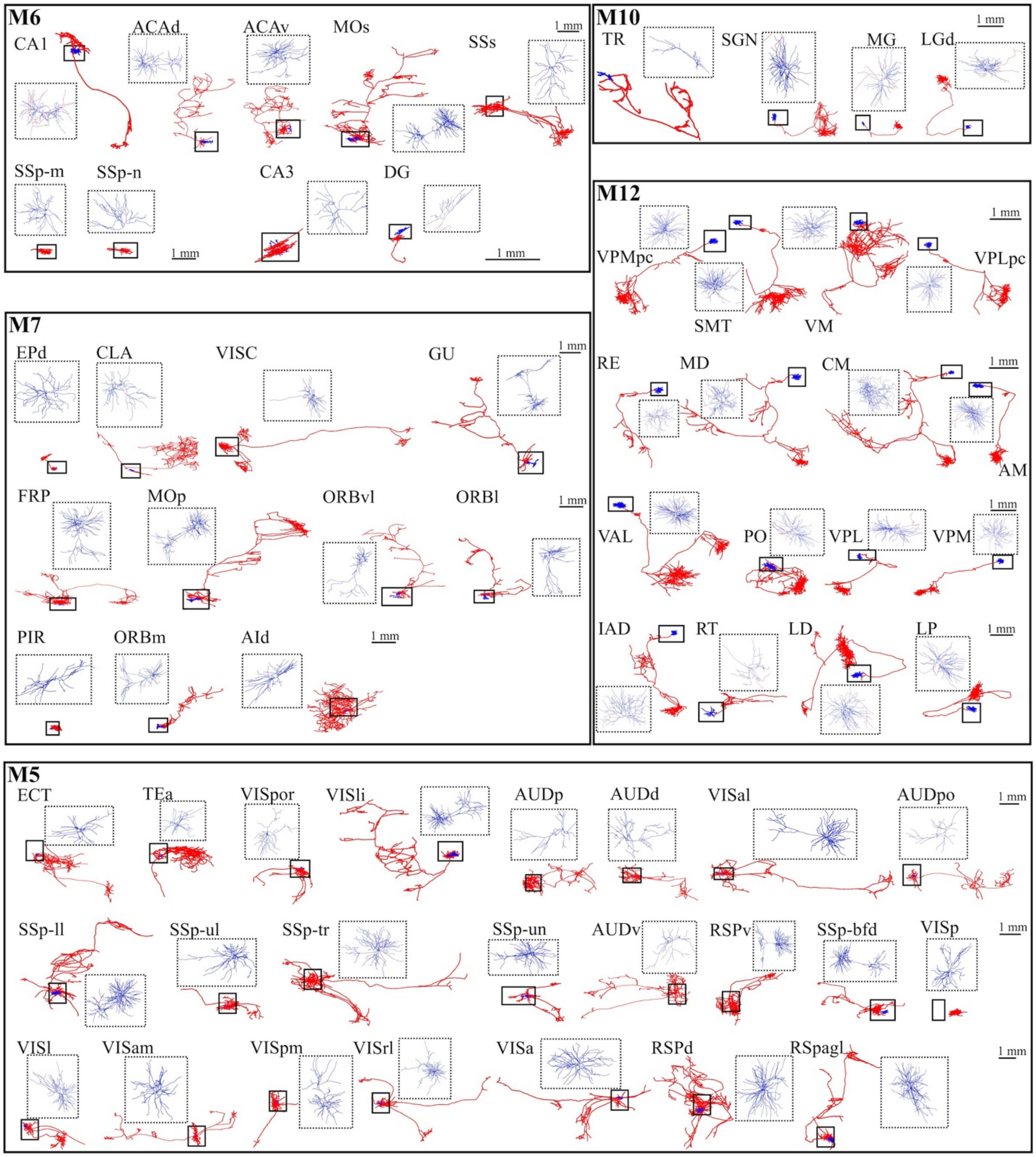

Supplementary Figure S24. **Cellular morphological diversity among modules.** Sagittal views of randomly selected single neuron morphologies within modules, estimated from neurite distribution as discussed in **Figure 2**. The dendrites of each neuron are highlighted within the dashed inset rectangle, colored in blue. The axons are shown using red lines. The respective neuron types are indicated adjacent to the morphologies. Scale bars are based on the single neuron morphologies in each row.

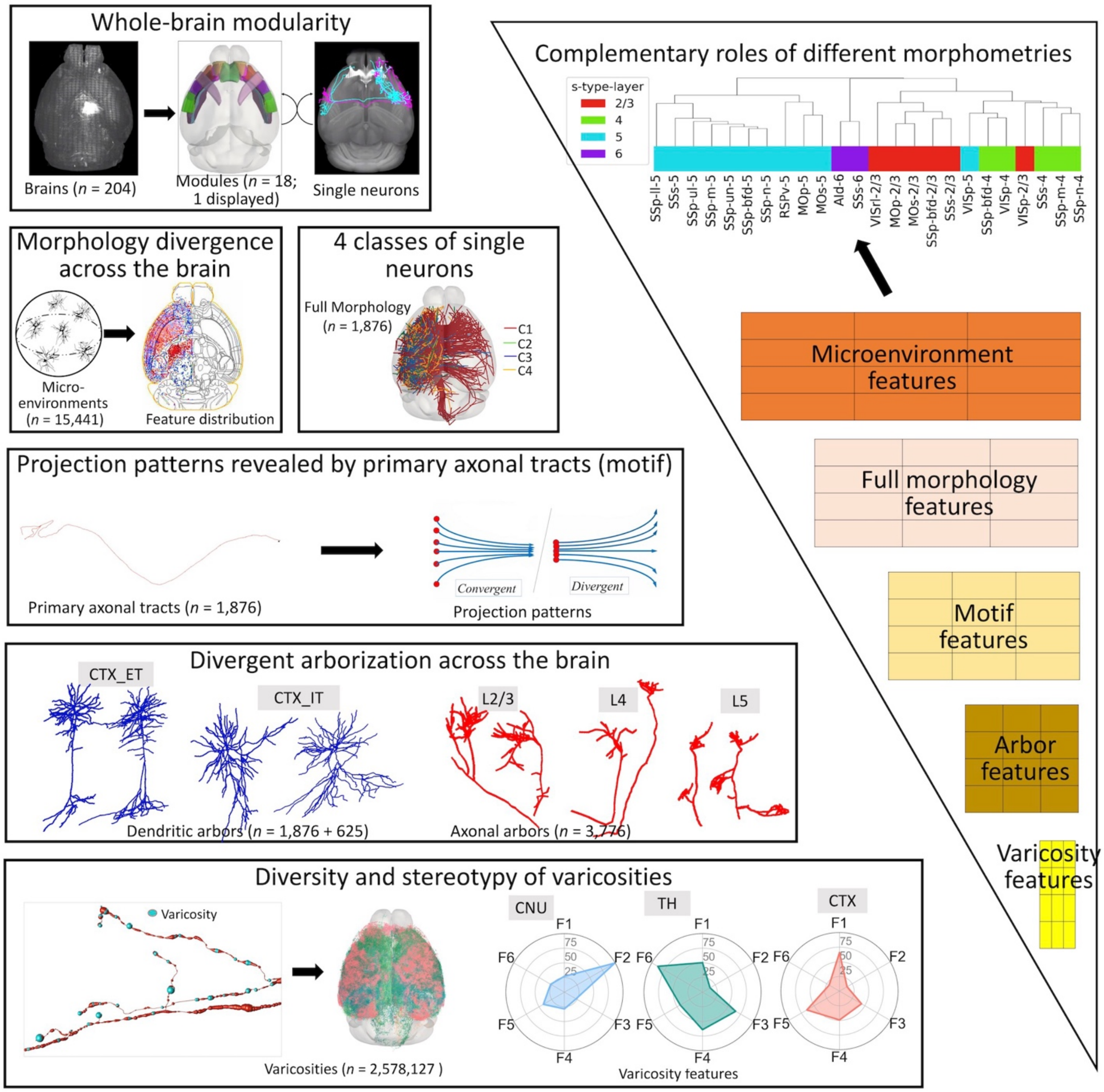

239
240 ***Supplementary Figure S25.*** **Schematic illustration of the main results.**
