# Supplementary material for: Neuronal diversity and stereotypy at multiple scales through whole brain morphometry
Source: Nat Commun. 2024 Nov 26;15:10269. doi: 10.1038/s41467-024-54745-6 (PMC11599929; doi:10.1038/s41467-024-54745-6)
Supplement: Supplementary file 2 — Description of Additional Supplementary Files [file 41467_2024_54745_MOESM2_ESM.pdf]

Supplementary Data 1 TableS1_SEU-ALLEN_brains_1223_204brains.xlsx A list of the 204 brains used in the study, comprising 190 fMOST brains, 10 STPT brains, and 4 LSFM brains, along with their respective meta information.

Supplementary Data 2 TableS2_soma_region314_counts.xlsx
Summarization of the total number of somas annotated for all CCF-R314 regions.

Supplementary Data 3 TableS3_Soma_morphometry_1222.xlsx
A complete list of all 182,497 somas annotated, along with their meta information, including their locations in both the original image space and CCFv3 atlas space, and the corresponding CCFv3 regions based on image registration.

Supplementary Data 4. TableS4_region_sets.xlsx
Correlated regions for each target region. Values in the "Type" column indicate whether all regions belong to the same compound area (intra-CA) or different compound areas (cross-CA).

Supplementary Data 5. TableS5_modules.xlsx
To avoid duplication, only the upper triangular values of pairwise correlation coefficient matrix are shown.

Supplementary Data 6. TableS6_Full_morphometry_1222.xlsx
The metadata for SEU-A1876, encompassing information regarding their brain sources, coordinates in the original image space and the CCFv3 space, the regions in which their somas are situated, and their respective projection types.

Supplementary Data 7. TableS7_teminologies.xlsx
Explanations of terminologies used in this work.

Supplementary Data 8. TableS8_neuroanatomical_novelty_table.pdf
Key novelties of this work.
