# Supplementary material for: Neuronal diversity and stereotypy at multiple scales through whole brain morphometry
Source: Nat Commun. 2024 Nov 26;15:10269. doi: 10.1038/s41467-024-54745-6 (PMC11599929; doi:10.1038/s41467-024-54745-6)
Supplement: Supplementary file 10 — Supplementary Data 8 [file 41467_2024_54745_MOESM10_ESM.pdf]

Level	Morphology type	Scale	Morpho-features	Whole-brain registration needed?	Data	Previous knowledge	Previous major literatures	Our key findings	Major location of our results in Figures / Tables	Our data sharable?	Shared results data volume / size
Whole brain	Cross-scale	1μm - 10000+ μm	Multiscale features from neuron-population to axonal varicosity	Yes	Petavoxels	NO existing systematic cross-scale study	NO	1. Highly-modularized neuroanatomical organizations are observed in the central nervous system (CNS) neurons, with respect to brain regions, cortical laminations, and projection types of cortical neurons.<br>2. Morphometry at different scales complements the classification of neurons in the central nervous system.	Figure 1; Figure 8	Yes	3.7 Petavoxels
Neuron-population (global)	Neuron-population	1000μm-10000μm	Number of neurite voxels across all brain regions	Yes	Petavoxels	Some whole-brain modularity of mammalian brains have been analyzed at macro- or meso-scales	1. Bertolero et al., PNAS, 2015, doi: 10.1073/pnas.1510619112<br>2. Wang et al., J Neurosci, 2012, doi: 10.1523/JNEUROSCI.6063-11.2012<br>3. Oh et al., Nature, 2014, doi: 10.1038/nature13186	We identified 18 new modules from the complete set of 314 brain regions at a submicron resolution.	Figure 2D; Supplementary Table S5	Yes	3.7 Petavoxels
Neuron population (local)	Dendritic microenvironment	30 million μm³	Spatially enhanced morphological features	Yes	Thousands to millions microenvironments	Parcellations reported in low resolution, incapable of differentiating subregions that containing neurons with different morphology and function.	1. Lein et al., Nature, 2007, doi: 10.1038/nature05453<br>2. Oh et al., Nature, 2014, doi: 10.1038/nature13186<br>3. Dong et al., Wiley, 2008<br>4. Paxinos et al., Elsevier, 2001, 2012<br>5. Wang et al., 2020, doi: 10.1016/j.cell.2020.04.007	Microenvironment representation of neighoring neurons discriminates sub-regional parcellation	Figure 3	Yes	15441
Single-neuron	Full morphology	500μm-5000μm	Spatially enhanced Morphological features	Yes	Hundreds to thousands neurons	Single neurons could not be classified into discrete clusters, based on conventional features of projection or morphology.	1. Peng et al., Nature, 2021, doi: 10.1038/s41586-021-03941-1<br>2. Winnubst et al., Cell, 2019, doi: 10.1016/j.cell.2019.07.042	We identified 4 discrete cross-areal single neuron clusters from 1,876 single neurons, covering 94 brain regions. This is the first time such clear clustering has been shown, and is only possible with our enhanced analysis	Figure 4	Yes	1876
Sub-neuronal	Sub-neuronal arbor	100μm-2000μm	Morphological features (e.g., volume and path length)	No for sub-areas, yes for comparative studies	Thousands arbors	NO previous study	NO	Neuronal arborization pattern characterizes cortical, striatal, and thalamic neurons in mouse.	Figure 5	Yes	3776
	Primary axonal tracts	100μm-2000μm	Volume, length, and coordinates	Yes	Hundreds to thousands tracts	Projection patterns were experimentally estimated through various labeling techniques or transcriptome profiling, however quantifying the relationship between sources (somas) and targets (terminal axons) has been challenging due to their imbalanced scales.	1. Li et al., Brain Structure and Function, 2021, doi:10.1007/s00429-021-02289-6; Oh et al., Nature, 2014, doi: 10.1038/nature13186<br>2. Fernández-Nogales, Advanced Science, 2023, doi:10.1002/advs.202200615<br>3. Wang et al., Neurosci Bull, 2021, doi: 10.1007/s12264-020-00616-1<br>4. Peng et al., Nature, 2021, doi: 10.1038/s41586-021-03941-1	Confirmed quantitatively the convergent projection patterns for CP neurons, and divergent patterns for cortical and thalamic neurons.	Figure 6	Yes	1876
	Axonal varicosity	1μm-3μm	Customized features (see Figure 1E)	No for sub-areas, yes for comparative studies	Millions [predicted] boutons	Comparative analyses of the spatial preference of varicosities across the whole brain have not been found, and the distribution of local varicosities was evaluated only in small datasets, never at the whole brain scale	1. Julian et al., PLoS Computational Biology, 2010, doi: 10.1371/journal.pcbi.1000711<br>2. Karube et al., J Neurosci, 2004, doi: 10.1523/JNEUROSCI.4814-03.2004	1. Stereotyped axonal varicosity features are observed among neurons from the same brain structures (e.g., cortex, thalamus, and cerebral nuclei), while diverse across different structures.<br>2. Axonal branches successive to a varicosity-containing branch (near the soma) always contain varicosities	Figure 7	Yes	3.63 millions

Red color: key novelties. See the main text and Supplementary Figures / Tables for details. See video demos etc at https://sd-jiang.github.io/full_spectrum/
