# Supplementary material for: Neuronal diversity and stereotypy at multiple scales through whole brain morphometry
Source: Nat Commun. 2024 Nov 26;15:10269. doi: 10.1038/s41467-024-54745-6 (PMC11599929; doi:10.1038/s41467-024-54745-6)
Supplement: Supplementary file 11 — Reporting Summary [file 41467_2024_54745_MOESM11_ESM.pdf]

nature portfolio

Corresponding author(s): Hanchuan Peng

Last updated by author(s): Oct 25, 2024

Reporting Summary

Nature Portfolio wishes to improve the reproducibility of the work that we publish. This form provides structure for consistency and transparency in reporting. For further information on Nature Portfolio policies, see our Editorial Policies and the Editorial Policy Checklist.

Statistics

For all statistical analyses, confirm that the following items are present in the figure legend, table legend, main text, or Methods section.

n/a	Confirmed	
☐	☒	The exact sample size (*n*) for each experimental group/condition, given as a discrete number and unit of measurement
☐	☒	A statement on whether measurements were taken from distinct samples or whether the same sample was measured repeatedly
☐	☒	The statistical test(s) used AND whether they are one- or two-sided<br>*Only common tests should be described solely by name; describe more complex techniques in the Methods section.*
☒	☐	A description of all covariates tested
☒	☐	A description of any assumptions or corrections, such as tests of normality and adjustment for multiple comparisons
☐	☒	A full description of the statistical parameters including central tendency (e.g. means) or other basic estimates (e.g. regression coefficient) AND variation (e.g. standard deviation) or associated estimates of uncertainty (e.g. confidence intervals)
☐	☒	For null hypothesis testing, the test statistic (e.g. *F*, *t*, *r*) with confidence intervals, effect sizes, degrees of freedom and *P* value noted<br>*Give P values as exact values whenever suitable.*
☒	☐	For Bayesian analysis, information on the choice of priors and Markov chain Monte Carlo settings
☒	☐	For hierarchical and complex designs, identification of the appropriate level for tests and full reporting of outcomes
☐	☒	Estimates of effect sizes (e.g. Cohen's *d*, Pearson's *r*), indicating how they were calculated

Our web collection on statistics for biologists contains articles on many of the points above.

Software and code

Policy information about availability of computer code

Data collection	Single neuron reconstructions were annotated using Vaa3D (RRID:SCR_002609, version 3.601, https://github.com/Vaa3D).
Data analysis	The source codes are available via https://doi.org/10.5281/zenodo.13979929. Dependencies are summarized in the requirement.txt, which can be easily installed using the Python package manager, PIP. Detailed documentation and step-by-step instructions are provided in the repository. Video instructions showing how to use the package is available at https://sd-jiang.github.io/full_spectrum/.<br><br>Vaa3D (version 4.001) and Vaa3D-x (version 1.1.2) are available on the Vaa3D GitHub repository (https://github.com/Vaa3D) in both source code and released binary forms. Every version of Vaa3D encompasses core bindings such as TeraFly and TeraVR, plugins that include “Simple_Adaptive_Thresholding” filter, “global_neuron_feature”, Grayscale Image Distance Transform (GSDT), and auto-tracing algorithms like APP2, neuTube, and GD. The updated version of mBrainAligner can be found at https://github.com/Vaa3D/vaa3d_tools/tree/master/hackathon/mBrainAligner. MorphoHub is available at https://github.com/SD-Jiang/MorphoHub. Source codes for varicosity detection can be found at https://github.com/Vaa3D/vaa3d_tools/tree/master/hackathon/shengdian/BoutonDetection. The collaborative augmented reconstruction system (CAR) is available through https://github.com/neurogeom/CAR. The codes for full morphological feature extraction are accessible at https://github.com/Vaa3D/vaa3d_tools/blob/master/hackathon/shengdian/NeuroMorphoLib. Clustering and other machine learning algorithms, including K-Means, spectral clustering, HDBSCAN, and Principal Component Analysis (PCA), come from the third-party package scikit-learn (version 1.2.2) of Python (version 3.10). The Python derivative of mRMR (pymrmr, version 0.1.11, https://github.com/fbrundu/pymrmr) is used in this paper to select the most discriminating features. Hierarchical clustering in module detection and arbor analysis utilizes the hclust library from the stats package (version 4.2.2) in R (version 4.2.2).

For manuscripts utilizing custom algorithms or software that are central to the research but not yet described in published literature, software must be made available to editors and reviewers. We strongly encourage code deposition in a community repository (e.g. GitHub). See the Nature Portfolio guidelines for submitting code & software for further information.

Data

Policy information about availability of data

All manuscripts must include a data availability statement. This statement should provide the following information, where applicable:

- Accession codes, unique identifiers, or web links for publicly available datasets
- A description of any restrictions on data availability
- For clinical datasets or third party data, please ensure that the statement adheres to our policy

All morphometry data are accessible from both Zenodo (https://doi.org/10.5281/zenodo.13944322) and Google Drive (https://drive.google.com/drive/folders/1NwwTe840_0KQhv-zVLhw58LU9nntkb-F?usp=sharing). Documentation and video demonstrations for the dataset are available at https://sd-jiang.github.io/full_spectrum/. All brain images are generated from projects within the BICCN initiative, with most of them publicly accessible through the Brain Image Library (BIL) at https://www.brainimagelibrary.org/. The remaining 28 brain images will be made available shortly after compilation and uploading. Source data are provided with this paper.

Research involving human participants, their data, or biological material

Policy information about studies with human participants or human data. See also policy information about sex, gender (identity/presentation), and sexual orientation and race, ethnicity and racism.

Reporting on sex and gender	No human participants or related data and materials are used in our study.
Reporting on race, ethnicity, or other socially relevant groupings	No human participants or related data and materials are used in our study.
Population characteristics	No human participants or related data and materials are used in our study.
Recruitment	No human participants or related data and materials are used in our study.
Ethics oversight	No human participants or related data and materials are used in our study.

Note that full information on the approval of the study protocol must also be provided in the manuscript.

Field-specific reporting

Please select the one below that is the best fit for your research. If you are not sure, read the appropriate sections before making your selection.

☒ Life sciences ☐ Behavioural & social sciences ☐ Ecological, evolutionary & environmental sciences

For a reference copy of the document with all sections, see nature.com/documents/nr-reporting-summary-flat.pdf

Life sciences study design

All studies must disclose on these points even when the disclosure is negative.

Sample size	The number of mouse whole-brain images (204 brains, 3.7 Peta-voxels) was determined by the throughput of the imaging and the storage capacity we have. The number of single neuron reconstructions was determined by the throughput of manual annotations.
Data exclusions	All image datasets were utilized whenever possible. 1,741 reconstructions with manual classification were used for comparison across neuron classes or types, except for full morphology and axonal bouton analyses, which utilized all 1,876 reconstructions.
Replication	All attempts at replicating the results were successful using the data and codes we provided.
Randomization	Randomization was not applicable to this study because the analyses were applied to all datasets.
Blinding	Blinding is not applicable to the study design. There was no allocation of treatment and control groups.

Reporting for specific materials, systems and methods

We require information from authors about some types of materials, experimental systems and methods used in many studies. Here, indicate whether each material, system or method listed is relevant to your study. If you are not sure if a list item applies to your research, read the appropriate section before selecting a response.

Materials & experimental systems

n/a	Involved in the study
☒	☐ Antibodies
☒	☐ Eukaryotic cell lines
☒	☐ Palaeontology and archaeology
☒	☐ Animals and other organisms
☒	☐ Clinical data
☒	☐ Dual use research of concern
☒	☐ Plants

Methods

n/a	Involved in the study
☒	☐ ChIP-seq
☒	☐ Flow cytometry
☒	☐ MRI-based neuroimaging

Plants

Seed stocks	No plant or seed is used in this study.
Novel plant genotypes	No plant or seed is used in this study.
Authentication	No plant or seed is used in this study.
